# Microbiota perturbation by anti-microbiota vaccine reduces the colonization of *Borrelia afzelii* in *Ixodes ricinus*

**DOI:** 10.1186/s40168-023-01599-7

**Published:** 2023-07-24

**Authors:** Alejandra Wu-Chuang, Lourdes Mateos-Hernandez, Apolline Maitre, Ryan O. M. Rego, Radek Šíma, Stefania Porcelli, Sabine Rakotobe, Angélique Foucault-Simonin, Sara Moutailler, Vaidas Palinauskas, Justė Aželytė, Ladislav Sǐmo, Dasiel Obregon, Alejandro Cabezas-Cruz

**Affiliations:** 1 Anses, INRAE, Ecole Nationale Vétérinaire d’Alfort, UMR BIPAR, Laboratoire de Santé Animale, 94700 Maisons-Alfort, France; 2 Institute of Parasitology, Biology Centre, Czech Academy of Sciences, Ceske Budejovice, Czech Republic; 3 Faculty of Science, University of South Bohemia, Ceske Budejovice, Czech Republic; 4 Biopticka Laborator S.R.O, Plzen, Czech Republic; 5 Nature Research Centre, Akademijos 2, 09412 Vilnius, Lithuania; 6 School of Environmental Sciences, University of Guelph, Guelph, ON Canada

**Keywords:** Anti-microbiota vaccine, Vector microbiota, Lyme borreliosis, *Borrelia afzelii*, *Ixodes ricinus*

## Abstract

**Background:**

Ticks can transmit a broad variety of pathogens of medical importance, including *Borrelia afzelii*, the causative agent of Lyme borreliosis in Europe. Tick microbiota is an important factor modulating, not only vector physiology, but also the vector competence. Anti-microbiota vaccines targeting keystone taxa of tick microbiota can alter tick feeding and modulate the taxonomic and functional profiles of bacterial communities in the vector. However, the impact of anti-microbiota vaccine on tick-borne pathogen development within the vector has not been tested.

**Results:**

Here, we characterized the *Ixodes ricinus* microbiota modulation in response to *B. afzelii* infection and found that the pathogen induces changes in the microbiota composition, its beta diversity and structure of bacterial community assembly. Tick microbiota perturbation by anti-microbiota antibodies or addition of novel commensal bacteria into tick midguts causes departures from the *B. afzelii*-induced modulation of tick microbiota which resulted in a lower load of the pathogen in *I. ricinus*. Co-occurrence networks allowed the identification of emergent properties of the bacterial communities which better defined the *Borrelia* infection-refractory states of the tick microbiota.

**Conclusions:**

These findings suggest that *Borrelia* is highly sensitive to tick microbiota perturbations and that departure from the modulation induced by the pathogen in the vector microbiota pose a high cost to the spirochete. Network analysis emerges as a suitable tool to identify emergent properties of the vector microbiota associated with infection-refractory states. Anti-microbiota vaccines can be used as a tool for microbiota perturbation and control of important vector-borne pathogens.

Video Abstract

**Supplementary Information:**

The online version contains supplementary material available at 10.1186/s40168-023-01599-7.

## Background

Ticks are vectors of a multitude of pathogens that can cause infectious diseases of medical and veterinary importance. One major example of tick-borne disease is Lyme borreliosis [1], which is caused by a genospecies complex of the spirochete *Borrelia burgdorferi* sensu lato (s.l.) [2]. Among the genospecies, *Borrelia afzelii* is the causal agent of most cases of Lyme borreliosis in Europe [3]. *Borrelia* is maintained in nature owing to biological transmission mediated by ticks of the genus *Ixodes* [4]. *Borrelia* spirochetes are generally acquired by the larval or nymphal stages of ticks that feed blood on an infected vertebrate host [5]. Once ingested by the ticks, the spirochetes enter and colonize the gut. Following molting of the tick, at the next blood meal, *Borrelia* migrates from the gut to the salivary gland and are transmitted, by nymphs or adults, to a new host along with tick saliva [2, 3]. In this cycle of acquisition, colonization, and transmission of *Borrelia* spirochetes by ticks, the group of endogenous bacteria that form the tick microbiota might play a pivotal role on it.

Several studies have demonstrated that microbiota can shape the vector competence for pathogens in different arthropods [6–10]. In ticks, for example, the antibiotic-based disruption of the microbiota of *Dermacentor andersoni*, the Rocky Mountain wood tick, reduced the acquisition of the pathogen *Francisella novicida* [11]. Furthermore, the level of *F. novicida* was positively correlated with a decrease of *Francisella* endosymbionts quantity in the microbiota of *D. andersoni* demonstrating a positive relationship pathogen-endosymbiont [11]. Perturbation of *Ixodes scapularis* microbiota to a dysbiosic state reduced *B. burgdorferi *sensu stricto colonization in larvae [9]. *B. burgdorferi* abundance in *I. scapularis* ticks was negatively correlated with the abundance of some bacterial taxa such us *Pseudomonas* or *Staphylococcus* and positively correlated with *Sphingomonas* [12]. Associations between commensal bacteria and pathogen levels in ticks suggest intimate pathogen-microbiota interactions that could facilitate or limit pathogen colonization in the vector. Targeting specific bacteria of vector microbiota that facilitate pathogen colonization could be a possible method of control through transmission-blocking vaccines.

In general, experimental manipulation of the microbiota has been achieved by antibiotic exposure or sterile-rearing conditions of the vector. However, these methods induce global changes in the microbiota and make the depletion of specific bacteria difficult. Recently, anti-microbiota vaccines were proposed as a precise tool for microbiota manipulation [13, 14]. Notably, identification of the keystone taxon (i.e., highly connected taxa driving community composition and function), Enterobacteriaceae, and subsequent vaccination against it induced host antibodies that were ingested by the vector during the blood meal and correlated with a decreased abundance of Enterobacteriaceae in *Ixodes ricinus* microbiota [14]. Furthermore, anti-microbiota vaccine impacted tick physiology by increasing tick weight during feeding [13] and modulated tick microbiota composition and diversity in a taxon-specific manner [14]. The impact of anti-microbiota vaccines on pathogen development was shown in *Plasmodium relictum* and the mosquito vector *Culex quinquefasciatus* [15]. Immune targeting of vector-associated Enterobacteriaceae modulated *C. quinquefasciatus* microbiota composition and diversity and decreased the occurrence and abundance of *P. relictum* in the midguts and salivary glands of the mosquitoes [15].

In this study, we aim to test whether the manipulation of tick microbiota by anti-microbiota vaccination of host mice against the keystone taxon Enterobacteriaceae reduces *B. afzelii* colonization in the vector *I. ricinus*. Comparison of the uninfected tick microbiota with that exposed to *B. afzelii* infection, anti-microbiota antibodies, and a novel commensal bacterium allowed the identification of infection-permissive and infection-refractory states of the microbial communities. The results will inform novel interventions for the control of Lyme borreliosis and other vector-borne diseases.

## Materials and methods

### Ethics statement

In vivo experiments were performed at the Animal Facility of the Laboratory for Animal Health of the French Agency for Food, Environmental and Occupational Health & Safety (ANSES), Maisons-Alfort, France, according to French and International Guiding Principles for Biomedical Research Involving Animals (2012). The procedures were reviewed and approved by the Ethics Committee (ComEth, Anses/ENVA/UPEC), with animal experimentation permit number E 94 046 08.

### Mice and housing conditions

Six-week-old female C3H/HeN (Charles River strain code 025) mice were purchased from Charles River (Miserey, France) and kept for adaptation for 1 week before conducting experiments. During the study, mice were maintained in green line ventilated racks (Tecniplast, Hohenpeissenberg, Germany) at − 20 Pa, with food (Kliba nafaj, Rinaustrasse, Switzerland) and water ad libitum. The mice were kept at controlled room temperature (RT, 20–23 °C) and a 12-h (h) light: 12-h dark photoperiod regimen. The number of mice per cage was limited to five. Animals were monitored twice a day (d) by experienced technicians and deviations from normal behaviors or signs of health deterioration were recorded and reported.

### Bacterial cultures

Low passage *B. afzelii* CB43 were started from glycerol stocks and grown in Barbour-Stoenner-Kelly (BSK) -H (Sigma-Aldrich, St. Louis, MO, USA) media containing 6% rabbit serum and were kept at 33 °C for 7 days. *Escherichia coli* BL21 (DE3, Invitrogen, Carlsbad, CA, USA) was grown in Lysogeny Broth (LB, Sigma-Aldrich, St. Louis, MO, USA) at 37 °C under vigorous agitation overnight.

### Experimental infection of mice with B. afzelii

For *Borrelia* infection, 1 × 10^6^ *B. afzelii* CB43 in 250 µL of BSK-H media was injected subcutaneously (100 µL) and intraperitoneally (150 µL) into C3H/HeN mice. Control mice were injected with BSK-H media alone, following the same protocol as described before. Blood samples were collected from animals of all experimental groups, 3 weeks after inoculation to confirm the infection by western blot [16]. Additionally, the right ankle joint, the heart, and the skin were collected from each mouse of all experimental groups at the endpoint of the experiment to confirm the infection by qPCR (see below).

### Live bacteria immunization

Live bacteria vaccine was prepared using *E. coli* BL21 (DE3, Invitrogen, Carlsbad, CA, USA) as previously described [13]. Briefly, *E. coli* culture was washed with phosphate buffer saline (PBS) 10 mM NaH2PO4, 2.68 mM KCl, 140 mM NaCl, pH 7.2 (Thermo Scientific, Waltham, MA, USA), resuspended at 3.6 × 10^4^ colony-forming unit (CFU)/mL, and homogenized using a glass homogenizer. C3H/HeN mice were immunized subcutaneously with 100 µL of *E. coli* BL21 (1 × 10^6^ CFU per mouse) in a water-in-oil emulsion containing 70% Montanide™ ISA 71 VG adjuvant (Seppic, Paris, France), with a booster dose 2 weeks after the first dose. Control mice received a mock vaccine containing PBS and adjuvant.

### Tick infestation

**Fig. 1:**
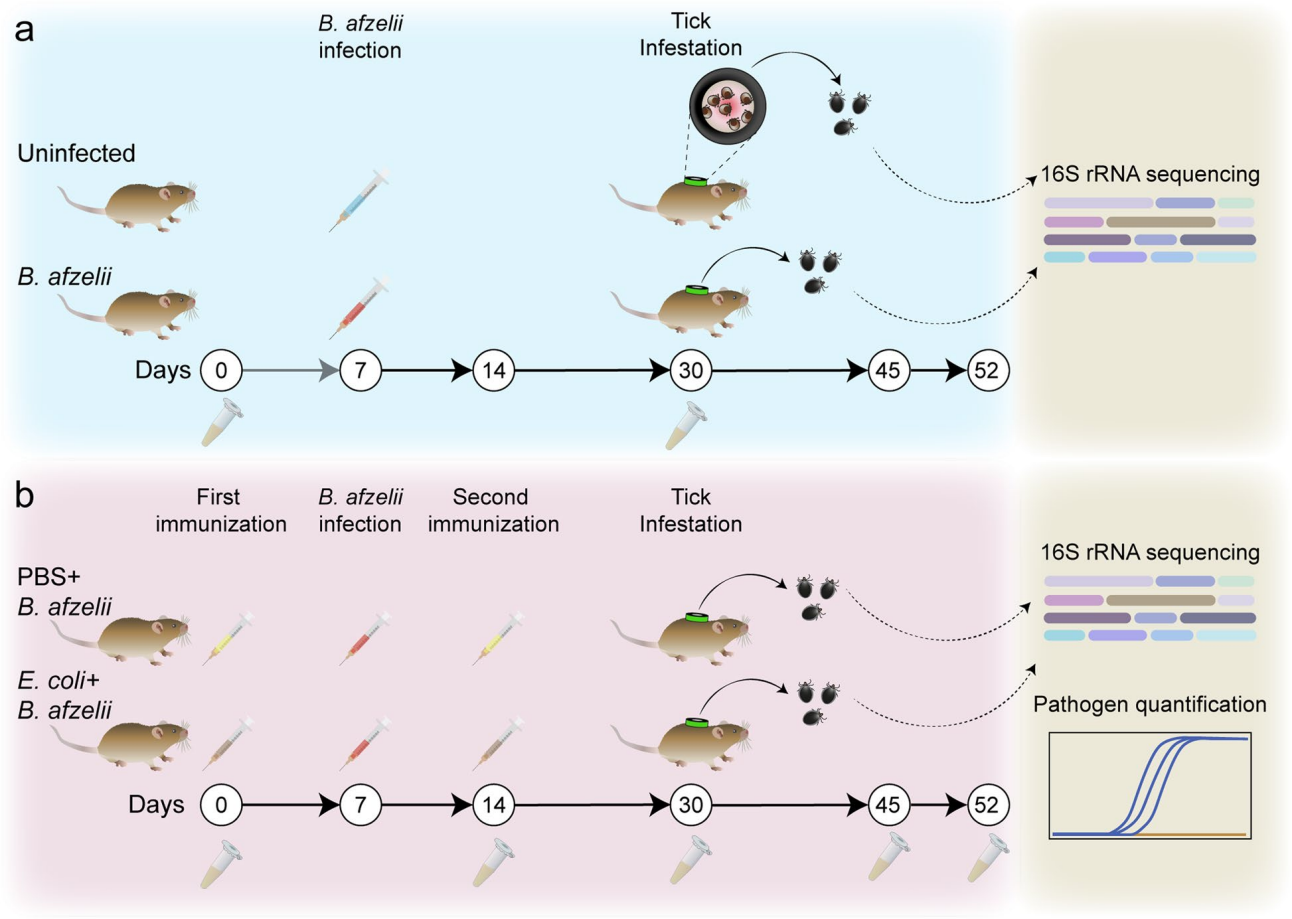
Experimental design and sample collection. **a** Mice were experimentally infected with spirochetes of *B. afzelii* grown in BSK-II medium (*n* = 10) while the uninfected group received an injection containing only BSK-II medium (*n* = 10). At day 30 (3 weeks post-infection), mice were infested with *I. ricinus* larvae (*n* = 100 per mouse). Sera of mice were rcollected to check infection by western blot and engorged ticks were collected and used for tick microbiota analysis. **b** Mice were immunized with a live vaccine containing *E. coli* BL21 (*n* = 5) or with a mock vaccine (PBS) (*n* = 5) at day 0. Subsequently, mice were experimentally infected with *B. afzelii* at day 7 followed by a booster shot of the live or mock vaccine at day 14. Mice from both groups were then infested with *I. ricinus* larvae (*n* = 100 ticks per mouse) at day 30. Mice sera were collected at different timepoints as indicated for ELISA experiments and engorged ticks were collected and their DNA extracted for tick microbiota analysis and pathogen level quantification

Pathogen-free unfed *I. ricinus* larvae were obtained from the colonies of UMR BIPAR, Maisons-Alfort, France. Mice were anesthetized by isoflurane and the 2-cm-outer-diameter EVA-foam capsule (Cosplay Shop, Brugge, Belgium) was glued on their shaved backs using non-irritating latex glue (Tear Mender, USA) as described in Mateos-Hernandez et al. [17]. Each mouse in the different groups was infested with one hundred *I. ricinus* larvae at day 30 (Fig. 1). The ticks, placed in a syringe, were deposited to the capsule by slowly pushing the plunger, and then, a plastic lid was used to close the capsule [17]. Tick feeding was visually monitored twice a day. Engorged larvae were collected in sterile tubes with holes and maintained with a light–dark (12 h/12 h) cycle in an incubator with > 97% relative humidity at 22 °C.

### Sera sample preparation

Blood samples obtained by retro-orbital bleeding were collected in sterile tubes on day 0 and day 30 in animals from all experimental groups. Additionally, blood samples were collected at days 14, 45, and 52 in mice from the PBS + *B. afzelii* and *E. coli* + *B. afzelii* groups. Blood samples were incubated for 2 h at RT, without anticoagulant, allowing for clotting, and then centrifuged at 5000 × *g* for 5 min at RT, twice. Sera were then separated and stocked in new sterile tubes at − 20 °C until use.

### Bacterial protein extraction

Lysates of *B. afzelii* culture were prepared to perform western blots. Seven milliliters of culture of *B. afzelii* with a density of at least 1 × 10^7^/mL was centrifuged at 8000 rpm for 10 min at 20 °C. The supernatant was then removed, and the bacterial pellet was washed twice with 1 mL cold HN-Buffer, centrifuged at 8000 rpm for 10 min at 20 °C. The resulting pellet was resuspended in 200 µL of bacterial protein extraction (B-PER) buffer (Thermo Scientific, Waltham, MA, USA) and incubated at RT for 10 min. The lysate was stored at − 20 °C until use. *Escherichia coli* protein extraction was done and later used for antigen coating for ELISA assay. *E. coli* culture were washed twice with PBS, centrifuged at 1000 × *g* for 5 min at 4 °C, resuspended in 1% Triton-PBS lysis buffer (Sigma-Aldrich, St. Louis, MO, USA), and homogenized with 20 strokes using a glass balls homogenizer. The homogenate was then centrifuged at 300 × *g* for 5 min at 4 °C, and the supernatant was collected. *B. afzelii* and *E. coli* protein concentration was determined using the Bradford Protein Assay (Thermo Scientific, San Jose, CA, USA) with bovine serum albumin (BSA) as standard.

### Western blot

Infectivity of *B. afzelii* in infected mice was checked by western blot using the sera of mice as primary antibodies. Lysates of *B. afzelii* were mixed with an equal volume of 2X Laemmli buffer (Thermo Scientific, Waltham, Massachusetts, USA) for a final quantity of 20 µg of protein/lane and were denatured by heat at 100 °C for 10 min. Prepared lysates were loaded in 4–15% Mini-PROTEAN TGX Stain-Free Protein gel (Bio-Rad, Hercules, CA, USA), and SDS-page electrophoresis was run then at 120 V for 1 h. Proteins were then transferred onto nitrocellulose membrane (Bio-Rad, Hercules, CA, USA) using semi-dry transfer method. Blotting was performed for 30 min at 25 V in a transfer cell (Trans-Blot SD, Bio-Rad, Hercules, CA, USA). The immunoblotting was done by blocking the membrane with 1% bovine serum albumin (BSA)/PBS (Sigma-Aldrich, St. Louis, MO, USA) for 2 h at RT, followed by incubation with mouse sera at a dilution of 1:100 in PBS at 4 °C overnight. The next day, membranes were washed in PBS three times for 10 min with gentle rocking. Then, membranes were incubated with HRP-conjugated antibodies (Abs, goat anti-mouse IgG) (Sigma-Aldrich, St. Louis, MO, USA) at 1:2000 dilution in PBS for 1 h at RT with gentle rocking. Membranes were washed three times and antibody detection was performed by chemiluminescence using Pierce ECL western blotting substrate (Bio-Rad, Hercules, CA, USA). Membranes were incubated with ECL reagent for 3 min, and membrane pictures were taken using ChemiDoc™ Touch Imaging System (Bio-Rad, Hercules, CA, USA).

### Indirect ELISA

The levels of Abs reactive against bacterial proteins were measured in mice sera as previously reported [13]. The 96-well ELISA plates (Thermo Scientific, Waltham, MA, USA) were coated with 100 µL per well of 0.5 µg/mL of *E. coli* BL21 protein extracts and incubated for 2 h at RT with gentle continual shaking at 100 rpm. Subsequently, plates were incubated overnight at 4 °C. The antigens were diluted in carbonate/bicarbonate buffer (0.05 M, pH 9.6). The next day, wells were washed three times with 100 µL of PBS containing 0.05% (vol/vol) Tween 20 (PBST), and then blocked by adding 100 µL of 1% human serum albumin (HSA)/PBS for 1 h at RT and gentle continual shaking at 100 rpm. After three washes, sera samples, diluted 1:700 in 0.5% HSA/PBS, were added to the wells and incubated for 1 h at 37 °C at 100 rpm. The plates were washed three times and 100 µL per well of HRP-conjugated Abs (goat anti-mouse IgG and IgM) (Sigma-Aldrich, St. Louis, MO, USA) was added at 1:1500 dilution in 0.5% HSA/PBST and incubated for 1 h at RT at 100 rpm. The plates were washed three times and the reaction was developed with 100 µL ready-to-use TMB solution (Promega, Madison, WI, USA) at RT for 20 min in the dark, and then stopped with 50 µL of 0.5 M H_2_SO_4_. Optimal antigen concentration and dilutions of sera and conjugate were defined using a titration assay. The optical density (OD) was measured at 450 nm using an ELISA plate reader (Filter-Max F5, Molecular Devices, San Jose, CA, USA). All samples were tested in triplicate, and the average value of three blanks (no Abs) was subtracted from the reads. The levels of Abs in pre-adsorbed sera were measured by coating with 100 µL per well of 0.5 µg/mL of *B. afzelii* protein extracts and following the above-described protocol.

### Tick capillary feeding

Capillary feeding was carried out using unfed *I. ricinus* nymphs. Glass capillary tubes of 3.5″ (Drummond Scientific, Broomall, PA, USA) were filled with a solution containing 5 × 10^6^ spirochetes of *B. afzelii* in BSK-II alone or combined with 2.5 × 10^7^ cells/mL of *E. coli* BL21. The proportion of *E. coli* BL21 to *B. afzelii* (5:1) was selected based on the abundance found for both bacteria in the uninfected group. Ticks were fixed on plastic Petri dishes with a double-sided adhesive tape and the filled capillary tubes were placed over ticks’ mouthparts. Ticks were left to feed for 4 h in a humidity chamber at 33 °C. After feeding, ticks were detached from the double-sided tape, collected in a sterile tube with holes and maintained in an incubator with > 97% relative humidity at room temperature for 6 h prior DNA extraction.

### Tick microinjection

Microinjection experiment was carried out in unfed nymphs. Microinjection capillaries 3.5″ (Drummond Scientific, Broomall, PA, USA) were fabricated by heating and pulling 1-mm glass capillary tubes in a glass micropipette puller device (P-1000 Sutter Instrument, Novato, CA, USA). For novel commensal bacteria addition experiment, a culture of *B. afzelii* grown in BSK-II media (5 × 10^6^ spirochetes/mL) alone or combined with *E. coli* BL21 (2.5 × 10^7^ cells/mL) was used for microinjection. For adsorption experiment, sera of *E. coli*-immunized and *B. afzelii*-infected mice were preincubated overnight at 4 °C with 250 ng/µL of *B. afzelii* proteins. The next day, the pre-adsorbed sera were centrifuged at full speed for 30 min and supernatant was recovered. Then, a culture of *B. afzelii* grown in BSK-II media (5 × 106 spirochetes/mL) alone or combined with PBS + *B. afzelii*, *E. coli* + *B. afzelii*, or pre-adsorbed *E. coli* + *B. afzelii* mouse sera were used for microinjection. Ticks were temporarily immobilized on a double-sided tape and microinjections were performed by micro-syringe pump (Drummond) connected to a Micro4 Controller (World Precision Instruments). A volume of 8 nl was injected into the anal pore of unfed nymphs. Microinjected ticks were incubated at room temperature for 2 or 6 h in an incubator at > 97% relative humidity prior to DNA extraction.

### DNA extraction

Genomic DNA was extracted from fully engorged larvae, nymphs, and mouse tissues. DNA from individual fully engorged larvae were extracted 15 days after the feeding. DNA from individual nymphs were extracted at the end of the period of incubation from the capillary feeding and microinjection experiments. DNA from mice tissues were extracted at the endpoint (day 52) of the experiment. Individual ticks were crushed with disposable probe while mice tissues were crushed with glass beads using a Precellys24 Dual homogenizer (Bertin Technologies, Paris, France) at 5500 × *g* for 20 s two times. Genomic DNA was extracted from tick and mouse tissues using a Nucleospin tissue DNA extraction Kit (Macherey–Nagel, Hoerdt, France). Each DNA sample from ticks and mouse tissue was eluted in 20 and 50 µL of sterile water, respectively. Genomic DNA quality (OD260/280 between 1.8 and 2.0) was measured with NanoDrop™ One (Thermo Scientific, Waltham, MA, USA).

### RNA extraction and cDNA synthesis

Total RNA was extracted from different mice tissues using Trizol reagent (DE3, Invitrogen, Carlsbad, CA, USA) following the manufacturer’s recommendations. The obtained RNA was reverse transcribed to cDNA using Superscript III (DE3, Invitrogen, Carlsbad, CA, USA) and was used for pathogen detection by RT-qPCR.

### Detection and quantification of B. afzelii load by PCR and qPCR

For the detection of *B. afzelii* in whole larvae or in mice tissues, a pre-amplification step was performed to improve pathogen DNA or cDNA detection. For that, total DNA or cDNA was pre-amplified using the PreAmp Master Mix (Fluidigm, CA, USA) according to the manufacturer’s instructions. Primers targeting the gene 23S rRNA for *Borrelia* spp. (23S rRNA-F ‘GAGTCTTAAAAGGGCGATTTAGT’, 23S rRNA-R ‘CTTCAGCCTGGCCATAAATAG’) were pooled by combining an equal volume of each primer for a final concentration of 200 nM. The reaction was performed in a final volume of 5 μL containing 1 μL Perfecta Preamp 5X, 1.25 μL pooled primer mix, 1.5 μL distilled water, and 1.25 μL DNA. The thermocycling program consisted of one cycle at 95 °C for 2 min, 14 cycles at 95 °C for 15 s and 4 min at 60 °C. At the end of the cycling program, the reactions were diluted 1:2 in Milli-Q ultrapure water. Subsequently, a quantitative PCR (qPCR) was carried out using the same aforementioned primers and an additional probe (23S rRNA-probe ‘AGATGTGGTAGACCCGAAGCCGAGT’) in a LightCycler 480 (Roche, Meylan, France). The reaction mixture contained 6 μL of FastStart universal probe master (Roche), 0.12 μL of 20 µM of primers 23S rRNA-F, 23S rRNA-R and TaqMan probe 23S rRNA-probe, 2 μL of pre-amplified DNA or cDNA sample, and Milli-Q ultrapure water up to 12 µL. The amplification program consisted of the following: 95 °C for 5 min, 45 cycles of 95 °C for 10 s, and 60 °C for 15 min. The spirochetes burden in ticks was obtained by interpolation of the CT value in a standard curve of “number of spirochetes vs CT” and then was normalized by the quantity of DNA in each sample.

### Detection of Enterobacteriaceae by PCR

DNA extracted from whole nymphal tick was used to detect Enterobacteriaceae using the following pair of primers: F-Enterobacteriaceae “ATGGCTGTCGTCAGCTCGT,” R-Enterobacteriaceae “CCTACTTCTTTTGCAACCCACTC” (from [18]) which target the 16S rRNA gene for Enterobacteriaceae. The reaction was performed in a final volume of 50 μL containing 5 μL 10X buffer, 4 μL of dNTP, 1 μL of each primer, 0.25 μL of Taq polymerase (Takara, Shiga, Japan), 1 μL DNA, and 37.75 μL of distilled water. The mixtures were amplified for 40 cycles at 98 °C for 10 s, 55 °C for 30 s, and 72 °C for 1 min, with a final extension at 72 °C for 3 min in an automated thermal cycler (Perkin-Elmer Cetus, Gouda, The Netherlands). Aliquots containing 3 μL of each amplified product, 1 μL of gel loading buffer (Thermo Scientific, Waltham, MA, USA), and 2 μL of distilled water were electrophoresed in 1.0% (wt/vol) agarose gel, with a molecular size marker (Thermo Scientific, Waltham, MA, USA) in parallel. DNA from a culture of *E. coli* BL21 was used as a positive control. Electrophoresis in TAE (40 mM Tris–acetate, 1 mM EDTA) buffer (Lonza Biosciences, Basel, Switzerland) was performed at 90 V for 1.5 h. The gel was stained with GelGreen (Biotium, Fremont, CA, USA) and photographed under ultraviolet light illumination.

### Illumina library preparation and sequencing of the 16S rRNA gene

At least 400 ng of fully engorged larvae DNA at ≥ 20 ng/μL concentration was sent for amplicon sequencing of the bacterial 16S rRNA gene, which was commissioned to Novogene Bioinformatics Technology Co. (London, UK). Libraries were prepared with NEBNext® Ultra™ IIDNA Library Prep Kit (New England Biolabs, MA, USA). A single lane of Illumina MiSeq system was used to generate 251-base paired-end reads from the V4 variable region of the 16S rRNA gene using barcoded universal primers (515F/806R) in samples from larvae engorged in uninfected mice (*n* = 10), larvae engorged on *B. afzelii*-infected (*n* = 10), *E. coli*-immunized and *B. afzelii*-infected (*n* = 8), or mock-immunized and *B. afzelii*-infected (*n* = 10) mice. The raw 16S rRNA gene sequences obtained from tick samples were deposited at the SRA repository (Bioproject No. PRJNA870490).

### Controls, identification and removal of contaminants

Two extraction reagent controls were set in which the different DNA extraction steps were performed using the same conditions as for the samples but using water as template. DNA amplification was then performed on the extraction control in the same conditions as for any other sample. Possible contaminating DNA in samples for 16S rRNA gene sequencing was statistically identified with “decontam” package [19] using the “prevalence” method. The prevalence is defined as the presence or absence across sample and the method used compares the prevalence of each sequence feature in true samples to the prevalence in negative controls to identify contaminants. Then, contaminants were removed from the dataset before downstream microbiome analysis [19].

### Analysis of 16S rRNA gene amplicon sequences

The analysis of 16S rRNA gene sequences was performed using QIIME 2 pipeline (v. 2021.4) [20]. Using DADA2 software [21] implemented in QIIME2, 16S rRNA gene sequences were first demultiplexed and then quality trimmed based on the average quality per base of the forward and reverse reads. The total length was trimmed at 180 and 154 in forward and reverse reads, respectively. Consequently, reads were merged and chimeric variants were removed. The resulting representative sequences were taxonomically assigned using a pre-trained naïve Bayes taxonomic classifier [22] based on SILVA database version 132 [23] and the 515F/806R primer set. The resulting taxonomic data tables were collapsed at the genus level and taxa with less than 10 total reads and present in less than 30% of samples of each dataset were removed. The taxonomic data tables were used for network analysis and keystone taxa identification. For convenience, in this study we refer to the *Borrelia* genus as a single genus, in the sense presented by [24], and did not consider the division of the genus *Borrelia* into two genera: the amended genus *Borrelia* containing only the members of the relapsing fever *Borrelia*, and the genus *Borreliella* containing the members of the Lyme disease *Borrelia* (i.e., *B. burgdorferi* s.l. complex) [25]. This does not imply that we are taking any position on the current debate on this issue [26].

### Construction of bacterial co-occurrence networks, identification of keystone taxa and attack tolerance test

Co-occurrence network analyses were performed using the Sparse Correlations for compositional data (SparCC) method [27] implemented in R studio [28]. Taxonomic data tables were used to calculate the correlation matrix. Correlation coefficients with magnitude > 0.75 or <  − 0.75 were selected. Network visualization and calculation of topological features and taxa connectedness (i.e., number of nodes and edges, modularity, network diameter, average degree, weighted degree, clustering coefficient, and centrality metrics) were performed using the software Gephi 0.9.2 [29]. The robustness of co-occurrence networks was tested with an attack tolerance test using the package NetSwan for R [30]. For this, networks were subjected to systematic removal of nodes using a directed attack where nodes are removed in decreasing order of their betweenness centrality (BNC) value (i.e., number of times a node is found on the shortest path between other nodes).

### Comparative network analysis

Comparison of the similarity of the most central nodes between two networks was done with the package “NetCoMi” [31] in R studio using the read count taxonomic tables. “Most central” nodes are defined as those nodes with a centrality value above the empirical 75% quartile. The comparison returns Jaccard indexes for each of four local measures (i.e., degree, betweenness centrality, closeness centrality, eigenvector centrality) of the sets of most central nodes as well as for the sets of hub taxa between the two networks compared. Thus, the Jaccard index express the similarity of the sets of most central nodes as well as the sets of hub taxa between the two networks. Jaccard index of 0 indicates completely different sets while a value of 1 indicates equal sets of most central nodes or hub taxa between the compared networks [31].

### Statistical analysis

Taxonomic data table, which consisted of sequencing-read counts, was used as input of the R package “ALDEx2” [32], which performed centered log-ratio (clr) transformation for all features in all the samples. Taxa abundances were compared using the R package “DeSeq2” [33]. The number of shared direct neighbor of the reference taxon *Escherichia-Shigella* in the different experimental groups was visualized using Venn diagrams implemented in the online tool http://bioinformatics.psb.ugent.be/webtools/Venn/. Alpha and beta diversity of bacterial taxa were carried out on rarified ASV tables. The alpha diversity was explored using the Pielou’s evenness and Faith’s phylogenetic metrics. Differences in alpha diversity metrics between groups were tested using a Kruskal–Wallis test. Beta diversity was explored using the Jaccard similarity and the Weighted Unifrac measures and compared between the groups using a PERMANOVA test. Betadisper function in R was used to determine the dispersion of samples based on Bray–Curtis distance matrix, and an analysis of variance (ANOVA) test was used for comparison of the dispersion of the samples between the groups. For testing similarity of most central nodes, two *p*-values P(J ≤ j) and P(J ≥ j) for each Jaccard index, which represent the probability that the observed value of Jaccard index is “less than or equal” or “higher than or equal”, respectively, to the Jaccard value expected at random, were calculated. Differences in relative Ab levels (i.e., OD) between groups of immunized mice in the different time points were compared using two-way ANOVA with Bonferroni multiple comparison tests applied for individual comparisons. Differences in relative Ab levels after pre-adsorption with *B. afzelii* proteins were compared using one-way ANOVA with Tukey’s multiple comparisons test. Differences in pathogen load in groups of infected ticks that received pre-adsorbed sera compared to the control groups were analyzed using Kruskal–Wallis test. Cluster analysis of different samples was based on Jaccard distance matrix and was done using the package “Vegan” [34] in R using the Ward method. The unpaired non-parametric Mann–Whitney *U* test was used to compare the tick parameters (i.e., percentage of ticks that dropped naturally, percentage of larvae that molt into nymphs and tick mortality) and the load of *B. afzelii* in ticks between groups. The Kruskal–Wallis test with Dunn’s multiple comparisons test was used to compare the loss of connectivity when removing 5 to 7% of nodes among all the experimental conditions. The Mann–Whitney *U* test and Kruskal–Wallis test followed by Dunn’s multiple comparisons test were performed in the GraphPad 8 Prism software (GraphPad Software Inc., San Diego, CA, USA). Differences were considered significant when *p* < 0.05.

## Results

### B. afzelii modulates the tick microbiota

**Fig. 2:**
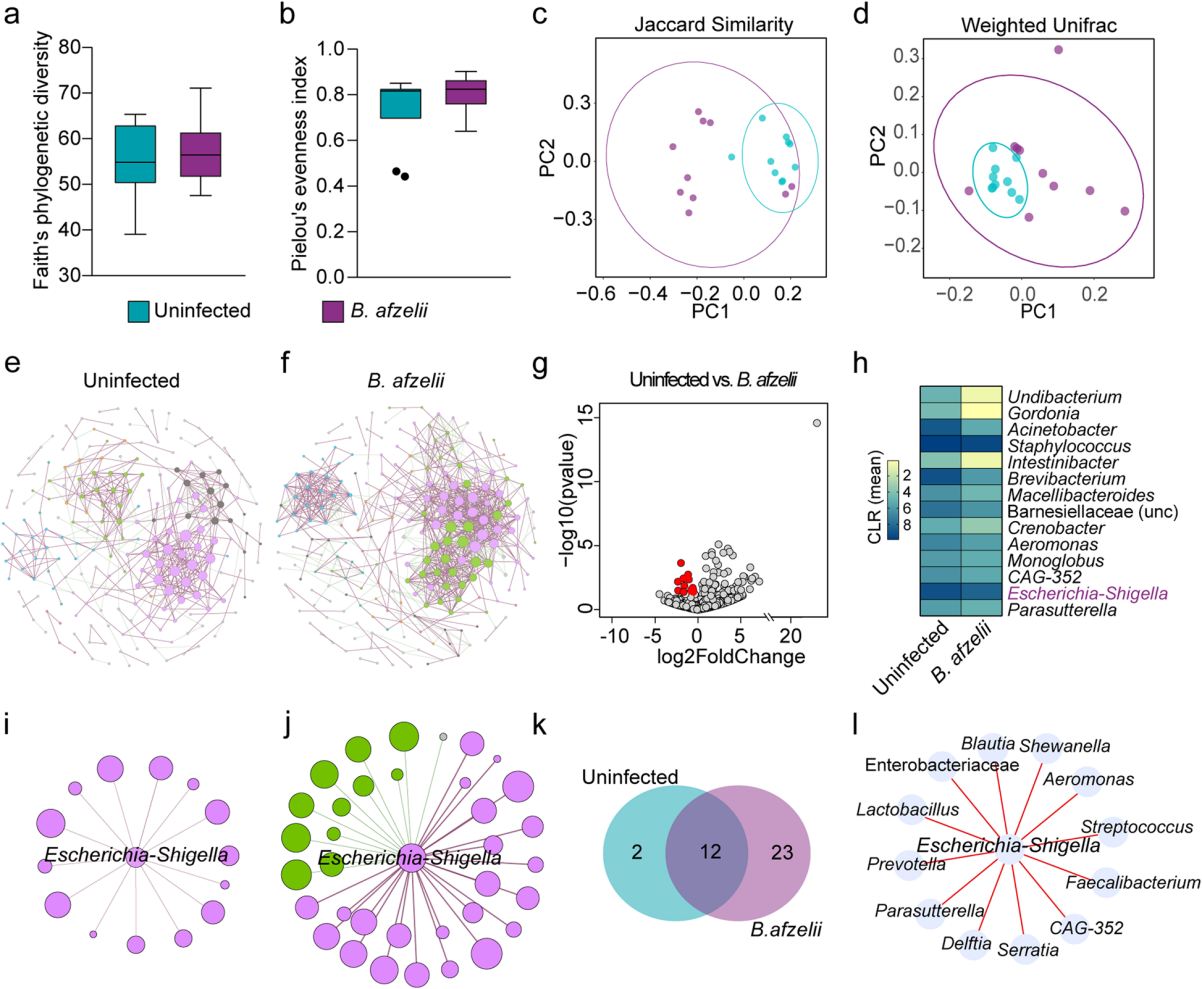
Impact of *B. afzelii* infection on tick microbiota diversity and community assembly. **a** Faith’s phylogenetic diversity and **b** Pielou’s evenness indexes were used to measure the richness and evenness, respectively, of microbiota of ticks fed on *B. afzelii*-infected and uninfected mice (Kruskal–Wallis, *p* > *0.05*). **c**, **d** Beta diversity of tick microbiota were analyzed with the **c** Jaccard and **d** Weighted Unifrac indexes to measure the similarity between the bacterial communities in the different experimental conditions (PERMANOVA, *p* < *0.05*). **e**, **f** Bacterial co-occurrence networks were inferred from 16SrRNA gene sequences obtained from ticks fed on **e** uninfected and **f** *B. afzelii*-infected mice. **g** Volcano plot showing the differential microbial abundance in tick microbiota from the uninfected and *B. afzelii* groups. Red points represent the taxa whose abundance are significantly lower in *B. afzelii* group compared to the uninfected one. **h** Heatmap representing the abundance (expressed as CLR) of the 14 taxa whose abundance were lower in *B. afzelii* group. **i**, **j** Sub-networks of the local connectivity of *Escherichia-Shigella* were extracted from the **i** uninfected and **j** *B. afzelii* co-occurrence networks. Nodes represent bacterial taxa and edges stand for co-occurrence correlation (SparCC > 0.75 or <  − 0.75). **k** Venn diagram showing the number of bacterial taxa that are common or unique among the neighbors directly connected to *Escherichia-Shigella* in the uninfected and *B. afzelii* groups. **l** Direction of associations of common direct neighbor to the taxon *Escherichia-Shigella* between the uninfected and *B. afzelii* groups. Red edges indicate positive co-occurrence associations in both groups. Rarified table of ASVs, used to measure the alpha and beta diversity, and taxonomic table were obtained from 16S rRNA gene sequences from ticks fed on uninfected mice (*n* = 10 individual larvae) and *B. afzelii*-infected mice (*n* = 10 individual larvae). Node size of co-occurrence networks or sub-networks is proportional to the eigenvector centrality value and node color is based on the modularity class. Thus, nodes with the same color belong to the same cluster. Positive and negative interactions between co-occurring bacteria are represented by the dark red and green edges, respectively. Only nodes with at least one connecting edge are displayed

To study the impact of *B. afzelii* infection on tick microbiota, *I. ricinus* larvae were fed on *Borrelia*-infected mice and uninfected mice (Fig. 1a). Subsequently, bacterial community composition and diversity of tick microbiota were analyzed using 16S rRNA gene profiling after statistical identification and removal of DNA features (Supplementary Table S1). Analysis of alpha diversity indexes showed that Faith’s phylogenetic diversity (Fig. 2a) as well as the evenness (Fig. 2b) did not differ between the ticks fed on *B. afzelii*-infected mice and the uninfected ticks (Kruskal–Wallis, *p* > 0.05). However, beta diversity analysis of tick microbiota revealed that *B. afzelii* infection led to a shift in the bacterial community composition and abundance, compared to the uninfected group, as measured using the Jaccard index (PERMANOVA, *F* = 1.84, *p* = 0.001, Fig. 2c) and Weighted unifrac distance (PERMANOVA, *F* = 2.34, *p* = 0.005, Fig. 2d), respectively. Furthermore, a permutation test for the evaluation of the homogeneity of dispersions based on Bray–Curtis distance matrix revealed no significant differences in the dispersion of the bacterial community between the two groups (*F* = 2.43, *p* > 0.05).

The impact of *B. afzelii* infection on the bacterial community assembly was assessed by construction of microbial co-occurrence networks. Visual inspection of the networks showed that infection with *B. afzelii* caused a shift in the bacterial community assembly patterns (Fig. 2e,f). Analysis of the topological features of the networks revealed an increased number of nodes and especially of edges in the microbial co-occurrence networks inferred from microbiota of ticks fed on *B. afzelii*-infected mice compared to the network of the uninfected group (Table 1). Similarly, the modularity and the average degree increased in the *B. afzelii* network compared to the uninfected one (Table 1). The observed Jaccard index for all the local centrality measures tested (i.e., degree, betweenness centrality, closeness centrality, eigenvector centrality, and hub taxa), except for the betweenness centrality, was higher than expected by random for the comparisons between the uninfected and *B. afzelii* networks (Supplementary Table S2), suggesting high similarity in the hierarchical organization of nodes in the two networks.

**Table 1:** Topological features of the microbial co-occurrence networks

Topological features	Experimental groups	
	**Uninfected**	***B. afzelii***
Nodes^a^	626 (208)^i^	645 (230)^i^
Edges^b^	474	1008
-Positives	386	688
-Negatives	88	320
Modularity^c^	0.908	1.476
Modules^d^	56	45
Network diameter^e^	11	11
Average degree^f^	1.514	3.126
Weighted degree^g^	0.771	0.977
Clustering coefficient^h^	0.409	0.568

^a^Nodes represent bacterial taxa with co-occurrence correlation SparCC > or <  − 0.75

^b^edges represent the number of connections/correlations

^c^modularity is the strength of division of a network into modules

^d^modules are sub-communities of bacteria that co-occur more frequently among each other than with other taxa

^e^network diameter is the shortest path between the two most separated nodes

^f^average degree is the average number of links per node

^g^weighted degree is the sum of the weight of all the edges connected to a node

^h^clustering coefficient is the degree to which nodes in a network tend to form clusters

^i^total nodes and nodes with at least one edge are inside brackets

Significant changes in the abundance of 65 taxa were found between ticks fed on *B. afzelii*-infected mice and the uninfected group (Wald test, *p* < 0.05, Supplementary Fig. S1). Taxon *Borrelia* changed significantly and its abundance was higher in the microbiota of ticks fed on *B. afzelii*-infected mice (Wald test, *p* = 0.02, Supplementary Fig. S1) compared to the uninfected group. Among the taxa whose abundance changed significantly, the abundances of 51 and 14 bacterial taxa increased and decreased respectively in ticks from *B. afzelii* group. The 14 taxa with significantly decreased abundance in *B. afzelii* group are represented in Fig. 2g,h.

Interestingly, the abundance of the keystone taxon *Escherichia-Shigella* decreased significantly in the *B. afzelii* group, compared with the uninfected group (Wald test, *p* = 0.03, Supplementary Fig. S1). The genus *Escherichia-Shigella* was described as a keystone taxon in the microbiome of *Ixodes* ticks [13, 14]. In addition, vaccine-induced antibodies specific to *Escherichia-Shigella* modulated the tick microbiota and induced changes on the tick physiology [13] and reduced vector competence of mosquitoes for *P. relictum* [15]. Visual inspection of the sub-networks of local interaction of *Escherichia-Shigella* showed that *B. afzelii* infection increased the number of direct neighbors co-occurring with *Escherichia-Shigella*, compared with the uninfected sub-network (Fig. 2i,j). Most of the nodes connected to the taxon *Escherichia-Shigella* in the uninfected sub-network were also present in the *B. afzelii* sub-network (Fig. 2k, Supplementary Table S3), and the type of connection between them (i.e., positive or negative correlation) was conserved (Fig. 2l). We also found 23 unique nodes in the *B. afzelii* sub-network, where 12 and 11 nodes have negative and positive co-occurrence correlations, respectively, with *Escherichia-Shigella*. Further characterization of the importance of the genus *Escherichia-Shigella* in the co-occurrence networks revealed an increased closeness centrality, betweenness centrality, and eigenvector centrality (Table 2). Notably, the betweenness centrality was the measure that changed the most where it increased six times in the network of ticks fed on *B. afzelii*-infected mice compared to those fed in uninfected mice. These results show that despite the abundance of *Escherichia-Shigella* is lower in *B. afzelii*-infected ticks, probably due to an increase in bacterial competition, the importance of this taxon increases in the networks. Altogether, the results showed that *B. afzelii* infection led to a shift in the tick microbiota characterized by changes in the beta diversity, bacterial abundance, some network properties, and the relative importance of *Escherichia-Shigella*.

**Table 2:** Centrality measures of the taxon *Escherichia-Shigella* in the uninfected and *B. afzelii* networks

Experimental groups	Closeness centrality^a^	Betweeness centrality^b^	Eigenvector centrality^c^
Uninfected	0.256	0.001	0.635
*B. afzelii*	0.350	0.006	0.903

^a^Closeness centrality indicates how close a node is to all other nodes in the network

^b^betweenness centrality indicates how much a given nodes is in-between others

^c^eigenvector centrality measures a node’s importance while giving consideration to the importance of its neighbors

### Anti-microbiota vaccine alters the tick microbiota shift induced by B. afzelii and decreases pathogen infection in ticks

**Fig. 3:**
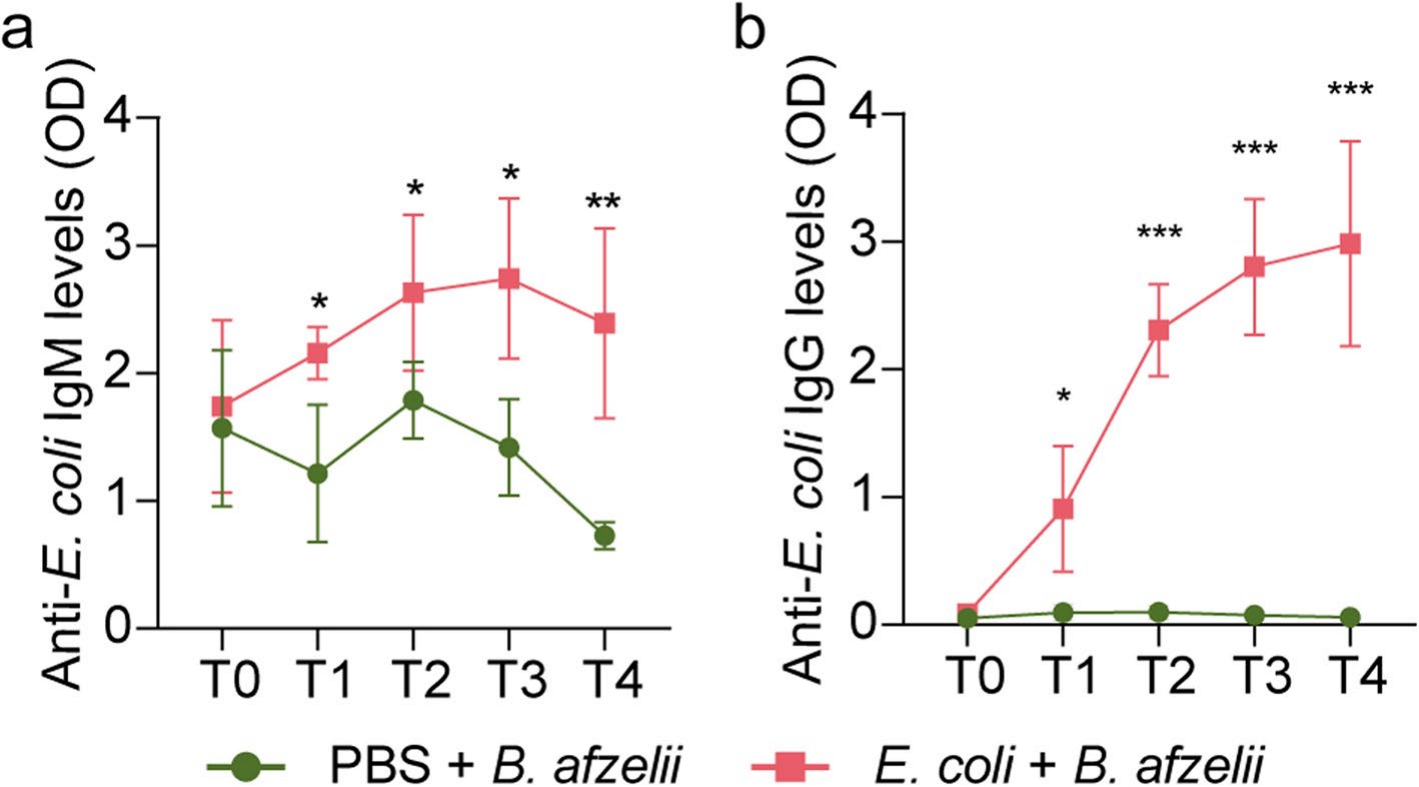
Antibody response of mice infected with *B. afzelii* and vaccinated with live *E. coli* or mock vaccine. The levels of **a** IgM and **b** IgG specific to *E. coli* proteins were measured by semi-quantitative ELISA in sera of *B. afzelii*-infected mice immunized with *E. coli* (pink) and a mock vaccine (green, PBS). Means and standard error values are shown. Results were compared by two-way ANOVA with Bonferroni test applied for comparisons between control and immunized mice. (* *p* < 0.05, ** p < 0.01; 1 experiment, *n* = 4 mice per experimental group and three technical replicates per sample)

The above results and previous evidence [9] led us to the hypothesis that altering the *Borrelia*-permissive states of the tick microbiota could alter pathogen colonization in the tick vector. To test this hypothesis, we altered the tick microbiota by targeting the keystone taxon *Escherichia-Shigella* [14] in *B. afzelii*-infected ticks and measured the impact on pathogen fitness. Immunization with an *E. coli*-based live vaccine was followed by experimental infection with *B. afzelii* and subsequent tick infestation on mice (Fig. 1b). *B. afzelii* infection was confirmed by qPCR (Supplementary Table S4a,b) and RT-qPCR (Supplementary Table S4c,d) in different mice tissues and by western blot using mice sera against *B. afzelii* protein extracts (Supplementary Fig. S2). Vaccination of mice with *E. coli* elicited an immune response where increased levels of antibodies IgM (Fig. 3a) and IgG (Fig. 3b) specific to *E. coli* was observed in mice sera compared to the control group, which received a mock vaccine. This immune response was maintained over time, at least 52 days after the first immunization (Fig. 3).

**Fig. 4:**
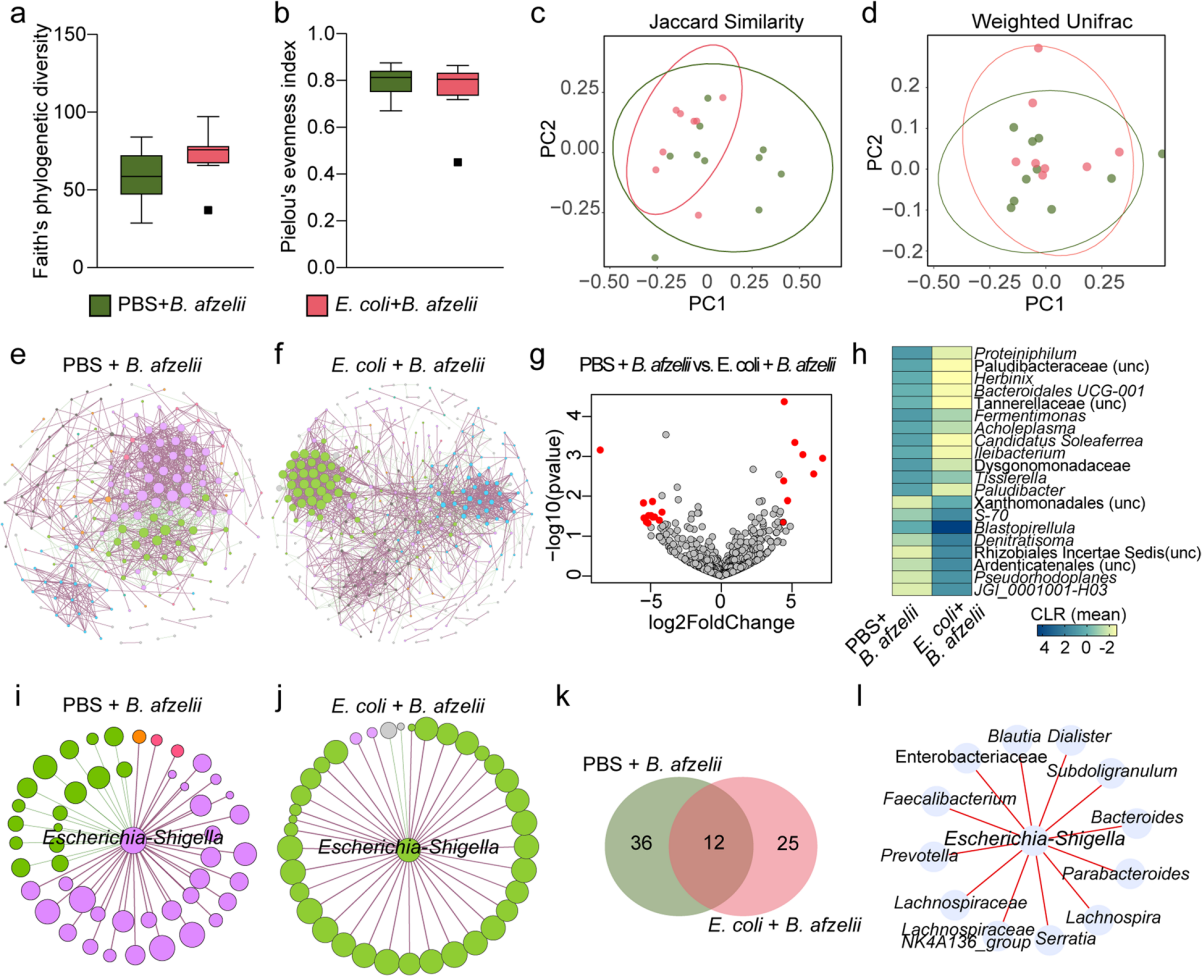
Impact of anti-microbiota vaccine and *B. afzelii* infection on tick microbiota diversity and community assembly. **a** Faith’s phylogenetic diversity and **b** Pielou’s evenness indexes were used to measure the richness and evenness, respectively, of microbiota of ticks fed on PBS + *B. afzelii*-infected and *E. coli* + *B. afzelii* mice (Kruskal–Wallis, *p* > 0.05). **c**, **d** Beta diversity of tick microbiota were analyzed with the **c** Jaccard and **d** Weighted Unifrac indexes to measure the similarity between the bacterial communities in the different experimental conditions (PERMANOVA, *p* > *0.05*). **e**, **f** Bacterial co-occurrence networks were inferred from 16SrRNA sequences obtained from ticks fed on **e** mock-immunized and *B. afzelii*-infected mice and **f** *E. coli*-immunized and *B. afzelii*-infected mice. **g** Volcano plot showing the differential microbial abundance in tick microbiota from the PBS + *B. afzelii*-infected and *E. coli* + *B. afzelii* groups. Taxa with highest differential abundance between the two groups are presented in red. **h** Heatmap representing the abundance (expressed as CLR) of the top 20 taxa with the highest absolute value of log2foldchange. **i**, **j** Sub-networks of the local connectivity of *Escherichia-Shigella* were extracted from the **i** PBS + *B. afzelii* and **j** *E. coli* + *B. afzelii* co-occurrence networks. **k** Venn diagram showing the number of bacterial taxa that are common or unique among the neighbors directly connected to *Escherichia-Shigella* in the PBS + *B. afzelii* and *E. coli* + *B. afzelii* groups. **l** Direction of associations of common direct neighbor to the taxon *Escherichia-Shigella* between the PBS + *B. afzelii* and *E. coli* + *B. afzelii* groups. Red edges indicate positive co-occurrence associations in both groups. Rarified table of ASVs, used to measure the alpha and beta diversity, and taxonomic table were obtained from 16S rRNA gene sequences from ticks fed on mock-immunized and *B. afzelii*-infected mice (*n* = 10 individual larvae) and *E. coli*-immunized and *B. afzelii*-infected mice (*n* = 8 individual larvae). Nodes of co-occurrence networks and sub-networks represent bacterial taxa and edges stand for co-occurrence correlation (SparCC > 0.75 or <  − 0.75). Node size is proportional to the eigenvector centrality value and node color is based on the modularity class. Thus, nodes with the same color belong to the same cluster. Positive and negative interactions between co-occurring bacteria are represented by the dark red and green edges, respectively. Only nodes with at least one connecting edge are displayed

Differences in the microbiota of ticks fed on mice infected with *B. afzelii* and immunized with the anti-microbiota or the mock vaccine were assessed by comparison of the alpha and beta diversity of the bacterial communities. Vaccination with *E. coli* had no significant impact on the bacterial diversity (Kruskal–Wallis, *p* > 0.05, Fig. 4a) or in the species evenness (Kruskal–Wallis, *p* > 0.05, Fig. 4b). Similarly, the beta diversity did not reveal a separate clusterization of the experimental groups as measured with the Jaccard index (PERMANOVA, *p* > 0.05, Fig. 4c) and weighted unifrac (PERMANOVA, *p* > 0.05, Fig. 4d).

Visual inspection of the microbial co-occurrence networks constructed from the microbiota of ticks fed on PBS + *B. afzelii* and *E. coli* + *B. afzelii* mice showed that the anti-microbiota vaccine modulated the bacterial community assembly (Fig. 4e,f), which was further confirmed by the topological features of these networks (Table 3). Specifically, the number of positive and negative edges increased notably in the *E. coli* + *B. afzelii* networks compared to the PBS + *B. afzelii* network (Table 3). Similarly, other topological features as the modularity, number of modules, the average degree, and network diameter increased in the *E. coli* + *B. afzelii* network compared to its control (Table 3). Testing the Jaccard index for the local network centrality measures revealed that the degree (Jacc = 0.389, *p* = 0.03) and the closeness centrality (Jacc = 0.404, *p* = 0.009) had Jacc values higher than expected by random for the comparisons between the two networks (Supplementary Table S5). Differential analysis of the abundance of each taxon in the microbiota of ticks showed that the abundance of 46 bacterial taxa changed significantly between ticks of the PBS + *B. afzelii* and *E. coli* + *B. afzelii* groups (Wald test, *p* < *0.05*, Supplementary Fig. S3). Specifically, the abundance of 23 bacterial taxa increased and also 23 taxa decreased in the microbiota of ticks fed on immunized and *B. afzelii*-infected mice compared to its control group. The top 20 taxa with the highest fold changes differences between the two groups are represented in Fig. 4g and are listed in Fig. 4h.

**Table 3:** Topological features of the microbial co-occurrence networks

Topological features	Experimental groups	
	**PBS + *B. afzelii***	***E. coli* + *B. afzelii***
Nodes	739(204)	735(378)
Edges	1124	2002
-Positives	867	1421
-Negatives	257	581
Modularity	0.963	1.1
Module	28	73
Network diameter	11	12
Average degree	3.042	5.448
Weighted degree	1.384	1.951
Clustering coefficient	0.590	0.471

To investigate if live bacteria immunization had an impact on the importance of *E. coli* in tick microbiome, sub-networks composed by the taxon *Escherichia-Shigella* and the direct neighbor were constructed. Comparisons of the sub-networks revealed that the number of co-occurring taxa with the taxon *Escherichia-Shigella* decreased in the networks of microbiota of ticks fed on *E. coli-*immunized and *B. afzelii*-infected mice (Fig. 4j), compared to those fed on mock-immunized and *B. afzelii*-infected mice (Fig. 4i). Comparison of the taxonomic identity of the direct neighbors between the PBS + *B. afzelii* and *E. coli* + *B. afzelii* sub-networks showed that the direct co-occurring taxa of the genus *Escherichia-Shigella* were mostly unique for each experimental condition and only 12 were shared between the sub-networks (Fig. 4k, Supplementary Table S6). A detailed comparison of the type of co-occurrence correlation between the taxon *Escherichia-Shigella* and the common taxa between the two sub-networks revealed that the type of connection was kept in the PBS + *B. afzelii* and *E. coli* + *B. afzelii* sub-networks (Fig. 4l). In contrast to the increase in the centrality measures of the taxon *Escherichia-Shigella* in the *B. afzelii* network compared to the uninfected group, we observed a decrease in all the three centrality measures (i.e., closeness, betweenness, and eigenvector centralities) in the microbiota of ticks fed on *E. coli-*immunized and *B. afzelii*-infected mice compared to those fed on mock-immunized and *B. afzelii*-infected mice (Table 4).

**Table 4:** Centrality measures of the taxon *Escherichia-Shigella* in the PBS + *B. afzelii* and *E. coli* + *B. afzelii* groups

Experimental groups	Closeness centrality	Betweeness centrality	Eigenvector centrality
PBS + *B. afzelii*	0.431	0.004	1
*E. coli* + *B. afzelii*	0.295	0.003	0.898

**Fig. 5:**
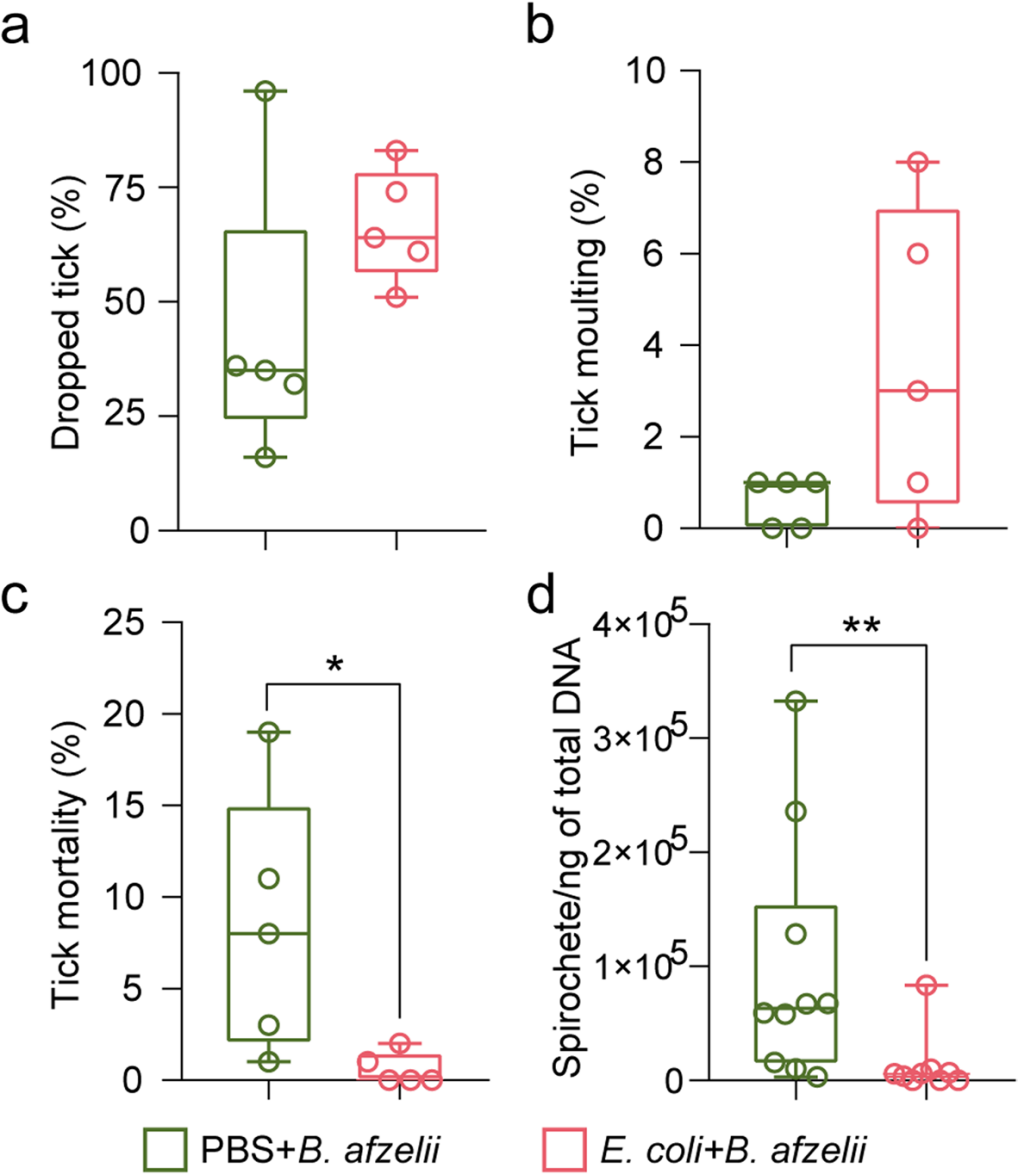
Impact of anti-microbiota vaccine on tick performance and *B. afzelii* development in infected ticks. **a** The percentage of ticks that dropped naturally after feeding, **b** the percentage of larvae molting into nymphs up to day 48, **c** the percentage of dead larvae, and **d** the load of spirochetes of *B. afzelii* were calculated and compared among the different experimental conditions. Each dot represents one mouse infested with 100 larvae. Means and standard deviation values are displayed. PBS + *B. afzelii*, *n* = 100 larvae/mouse; *E. coli* + *B. afzelii*, *n* = 100 larvae/mouse, *n* = 9–10 mice/condition. Mann–Whitney test, *p < 0.05, **p < 0.01

To test the impact of the anti-microbiota vaccine on tick physiology and pathogen fitness, several tick-performance parameters and *Borrelia* levels were compared between the PBS + *B. afzelii* and *E. coli* + *B. afzelii* groups. Results showed no significant differences in the percentage of ticks that dropped naturally (Fig. 5a) nor in the percentage of larvae that molt to nymphs (Fig. 5b) between the two groups. However, a significant decrease in the mortality of ticks from *E. coli* + *B. afzelii* group was observed compared to ticks from PBS + *B. afzelii* group (Mann–Whitney test, *p* = 0.02, Fig. 5c). Interestingly, the *B. afzelii* levels in ticks fed on *E. coli*-immunized and *B. afzelii*-infected mice were also significantly lower than in ticks fed on mock-immunized and *B. afzelii*-infected mice (Mann–Whitney test, *p* = 0.0056, Fig. 5d). Here we showed that anti-microbiota vaccines can disturb the tick microbiota and reduce *Borrelia* colonization, while previous studies showed that tick microbiota perturbation by sterile-rearing of ticks or exposure to antibiotics also reduced *Borrelia* colonization [9].

**Fig. 6:**
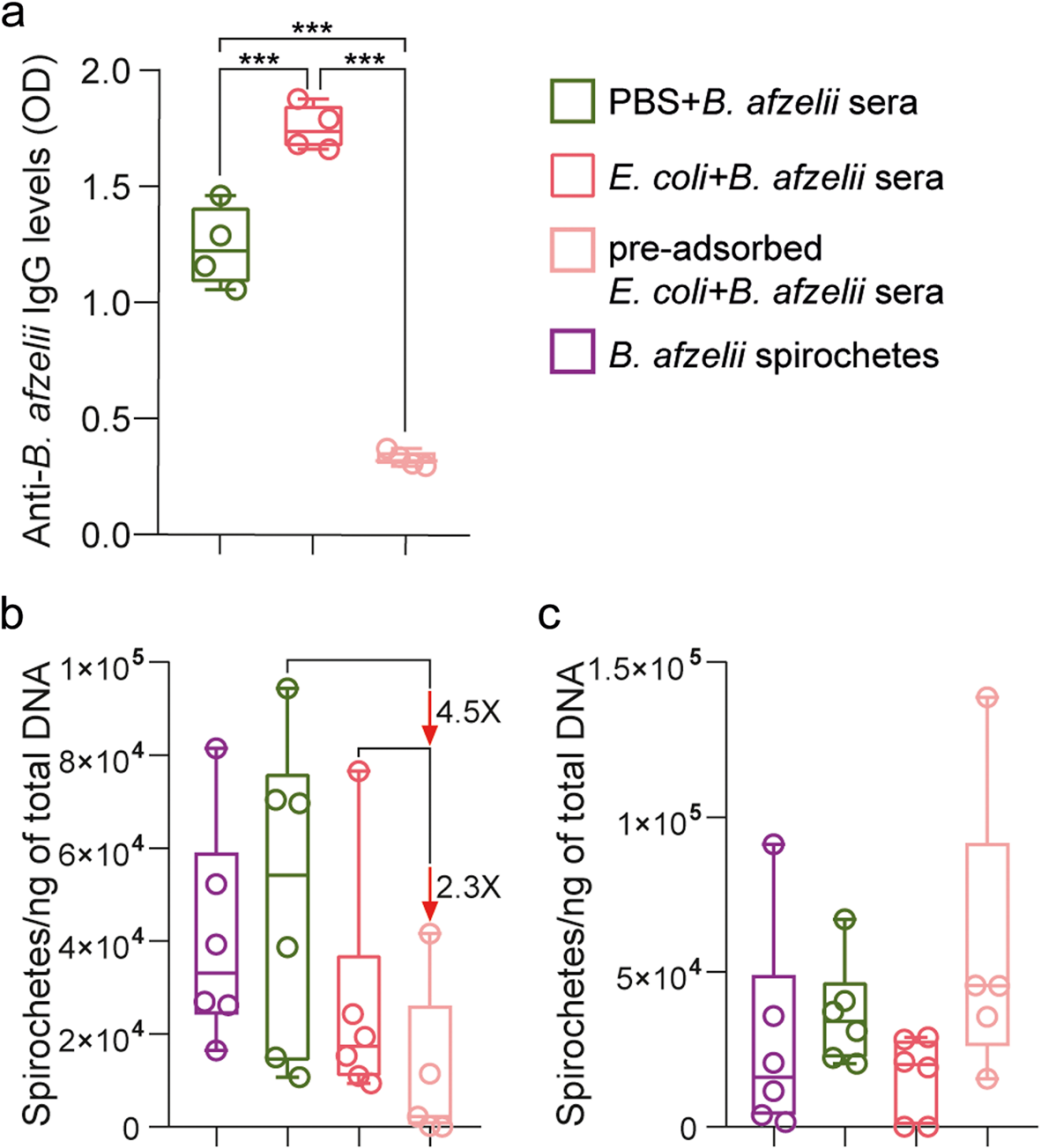
Impact of the removal of cross-reacting anti-*E. coli* antibodies on *B. afzelii* infection. **a** The levels of IgG specific to *B. afzelii* proteins were measured by semi-quantitative ELISA in pre-adsorbed sera of *B. afzelii*-infected mice immunized with *E. coli*, non-pre-adsorbed *E. coli* + *B. afzelii* or PBS + *B. afzelii* sera. One-way ANOVA with Tukey’s multiple comparisons test (*** p < 0.001; 4 technical replicates per sample). **b,c** The load of spirochetes of *B. afzelii* were measured in unfed nymphs microinjected with pre-adsorbed *E. coli* + *B. afzelii* sera combined with *B. afzelii* spirochetes and compared to nymphs that received *B. afzelii* spirochetes alone or combined with the non-pre-adsorbed *E. coli* + *B. afzelii*, PBS + *B. afzelii* sera. After microinjection, ticks were incubated for **b** 2 h or **c** 6 h. *n* = 6 nymphs/condition. Kruskal–Wallis test

Previous studies have shown a cross-reactivity of anti-*E. coli* antibodies against *Borrelia* antigens [35, 36]. To rule out the possibility that anti-microbiota vaccines reduce *Borrelia* load due to only the effect of the cross-reactivity of anti-*E. coli* antibodies, we removed antibodies anti-*E. coli* that could cross react with *B. afzelii* and study the impact of the remaining antibodies on pathogen load in ticks. We hypothesized that the remaining antibodies anti-*E. coli* will target *Escherichia* bacteria present in tick microbiota and influence the pathogen load. For that, we pre-adsorbed sera of *E. coli*-immunized and *B. afzelii*-infected mice with *B. afzelii* antigens to remove cross-reacting antibodies. Adsorption with *Borrelia* proteins reduced significantly the levels of antibodies recognizing *B. afzelii* in the *E. coli* + *B. afzelii* sera compared to non-pre-adsorbed *E. coli* + *B. afzelii* or PBS + *B. afzelii* sera (ANOVA, *p* < 0.05, Fig. 6a). Microinjection of pre-adsorbed *E. coli* + *B. afzelii* sera in the anal pore of infected ticks reduced 4.5 times and 2.3 times the load of *B. afzelii* compared to ticks microinjected with non-pre-adsorbed PBS + *B. afzelii* or *E. coli* + *B. afzelii* sera, respectively (Kruskal–Wallis, *p* > *0.05*, Fig. 6b) after 2 h of incubation. We observed, however, that these reductions of pathogen load in microinjected ticks were transient and the effect disappears after 6 h of incubation (Fig. 6c). These results suggest that although transiently, anti-*E. coli* antibodies produced by anti-microbiota vaccine reduced *Borrelia* load indirectly via tick microbiota modulation.

### Adding novel commensal bacteria reduces B. afzelii load in I. ricinus nymphs

To test whether other means of microbiota perturbation (i.e., addition of a novel commensal bacteria, [37–42]) also reduced *Borrelia* colonization, we used artificial capillary feeding and microinjection in the anal pore to introduce simultaneously *E. coli* BL21 and *B. afzelii* in *I. ricinus* nymph. By using *E. coli* BL21, a strain long kept in laboratory settings [43, 44], we ensured a novel interaction within the tick host, allowing us to rule out an evolutionary history between the added bacterium, the tick and the *Borrelia*, which may have reduced its impact in the microbiota.

Alpha and beta diversity of the bacterial community of tick that received artificially *B. afzelii* or *E. coli* + *B. afzelii* were analyzed using 16S rRNA gene profiling after statistical identification and removal of DNA features (Supplementary Table S7). Microbiota analysis showed that the simultaneous addition of *E. coli* and *B. afzelii* by microinjection (but not with capillary feeding) decreased significantly Faith’s phylogenetic diversity and the evenness (Kruskal–Wallis, *p* < 0.05, Supplementary Fig. S4a,b) of tick microbial community compared to the microbiota of ticks that received only *B. afzelii*. Furthermore, addition of *E. coli* + *B. afzelii* by microinjection or capillary feeding changed significantly the bacterial composition or abundance of tick microbiota compared to ticks that received *B. afzelii* (PERMANOVA, *p* < 0.05, Supplementary Fig. S4c,d).

**Fig. 7:**
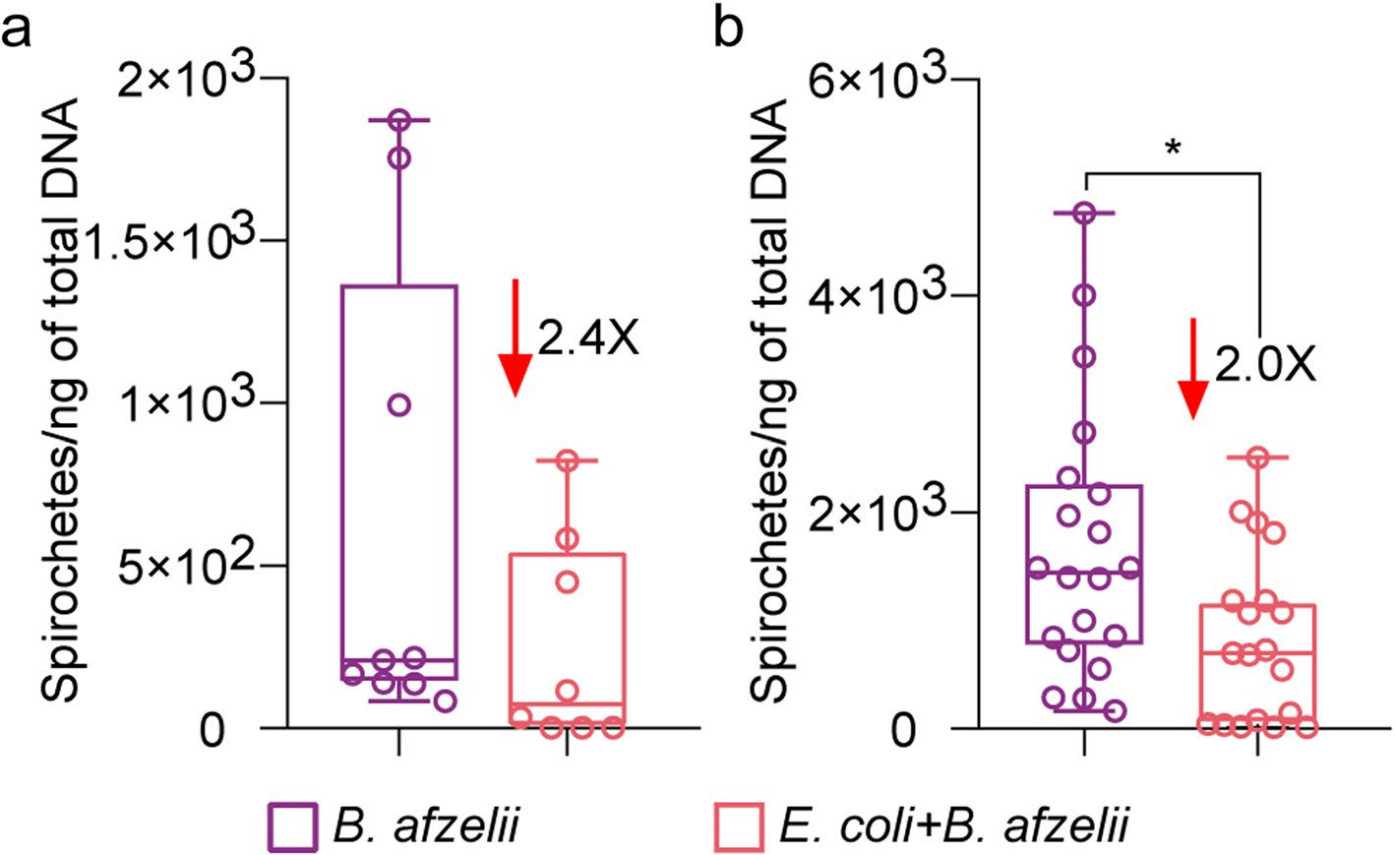
Impact of the introduction of *E. coli* on *B. afzelii* infection. The load of spirochetes of *B. afzelii* were measured in **a** *I. ricinus* nymphs fed artificially with *E. coli* + *B. afzelii* and in **b** nymphs injected with *E. coli* + *B. afzelii* in the anal pore. The results were compared to nymphs that received only *B. afzelii* spirochetes. Means and standard deviation values are displayed. *n* = 8–9 nymphs/condition for capillary feeding, *n* = 19–20 nymphs/condition for microinjection. Mann–Whitney test, *p < 0.05

After 6 h incubation, we compared the pathogen loads in ticks exposed to *E. coli* and *B. afzelii* with those exposed only to *B. afzelii* spirochetes. The presence of added *E. coli* in nymphs after capillary feeding or microinjection was confirmed by PCR (Supplementary Fig. S5). *B. afzelii* levels were 2.4 (Mann–Whitney test, *p ˃* 0.05, Fig. 7a) times lower in nymphs capillary fed with *E. coli* compared to nymphs fed only with *B. afzelii*. For nymphs microinjected with *E. coli* and *B. afzelii* and incubated for 6 h, *B. afzelii* load decreased 2.0 times compared to nymphs injected only with *B. afzelii* (Mann–Whitney test, *p* = 0.01, Fig. 7b).

This suggests that *Borrelia* is highly sensitive to tick microbiota perturbations and that deviations from the modulation induced by the pathogen in the vector microbiota pose a high cost to the spirochete.

### Defining B. afzelii infection-refractory states in the I. ricinus microbiota

**Fig. 8:**
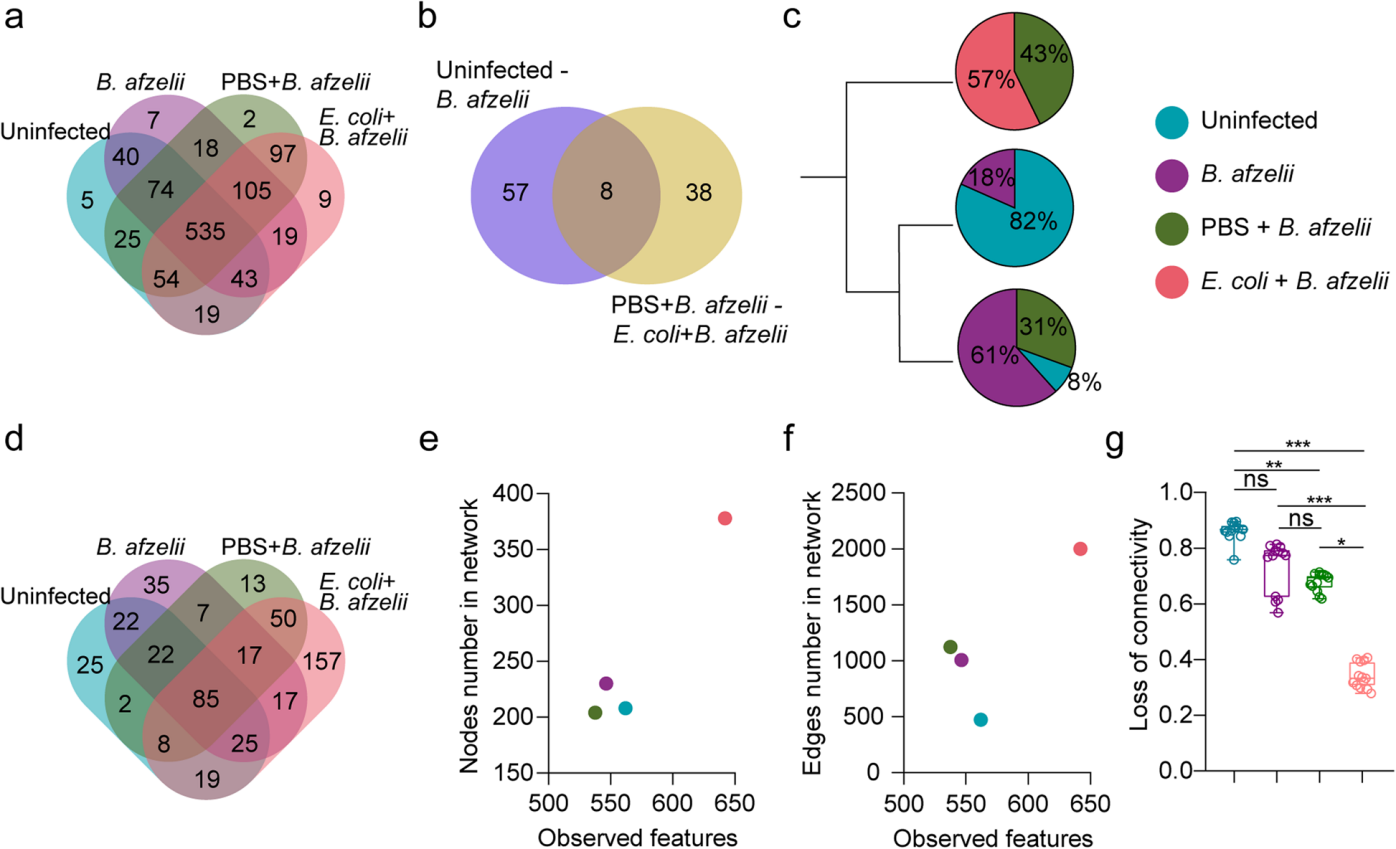
*B. afzelii* infection-refractory state of *I. ricinus* microbiota. Venn diagram showing **a** the common and unique bacterial taxa in tick microbiota for the uninfected, *B. afzelii*, PBS + *B. afzelii*, and *E. coli* + *B. afzelii* groups, **b** the shared and unique taxa whose abundance changed significantly between the uninfected-*B. afzelii* and PBS + *B. afzelii*-*E. coli* + *B. afzelii* comparisons. **c** Dendrogram of clustering for samples of tick microbiota from different experimental conditions. **d** Venn diagram showing the common and unique nodes found in microbial co-occurrence networks from all conditions. Scatter plot showing the mean of observed features versus number of **e** nodes and **f** edges found in the microbial co-occurrence networks and **g** Scatter plot showing the loss of connectivity when 5 to 7% of nodes are removed from the microbial co-occurrence network. Kruskal–Wallis test with Dunn’s multiple comparisons test, *p < 0.05, **p < 0.01, ***p < 0.001

A global comparison between the four different experimental conditions (i.e., uninfected, *B. afzelii*, PBS + *B. afzelii* and *E. coli* + *B. afzelii)* was performed aiming to define ecological properties of the infection-refractory states. We first compared the taxonomic profile among all the experimental groups and we found that a high number of bacterial taxa (i.e., 535 taxa) were shared among the 4 groups. Only 2 to 9 taxa were exclusively found in each experimental condition (Fig. 8a). When comparing the list of taxa whose abundance changed significantly between the uninfected and *B. afzelii* groups (Supplementary Fig. S1) and between the PBS + *B. afzelii* and *E. coli* + *B. afzelii* groups (Supplementary Fig. S3), we observed that only 8 taxa changed significantly their abundance in the uninfected-*B. afzelii* comparison as well as in the PBS + *B. afzelii* and *E. coli* + *B. afzelii* comparison. Interestingly, most of the taxa were unique for each set of comparisons, where 38 taxa were found exclusively in the comparison between the uninfected and *B. afzelii* groups and 57 in the comparison between the PBS + *B. afzelii* and *E. coli* + *B. afzelii* groups (Fig. 8b). Furthermore, cluster analysis of the microbiota based on the Jaccard index showed that the samples from different experimental conditions clustered in three different groups (Fig. 8c, Supplementary Fig. S6). Each cluster was formed mostly by tick microbiota samples from one experimental condition, namely *E. coli* + *B. afzelii*, uninfected and *B. afzelii* groups. Samples from PBS + *B. afzelii* clustered in two different groups, with samples from *B. afzelii* or *E. coli* + *B. afzelii* groups. It is noteworthy that all the samples of tick microbiota from the *E. coli* + *B. afzelii* group clustered in one separated group which is not the case for the other experimental conditions.

Subsequently, we compared networks emergent properties to determine their possible contribution to the *B. afzelii* infection-refractory state in tick microbiota. Comparison of the identity of the nodes that are involved in microbe-microbe interactions showed that 85 bacterial taxa are common in the networks of all experimental groups (Fig. 8d). Interestingly, the network that presented the highest number of unique taxa (i.e., 157 nodes) was the one inferred from *E. coli* + *B. afzelii* group compared to the uninfected (i.e., 22 nodes), *B. afzelii* (i.e., 35 nodes), PBS + *B. afzelii* (i.e., 13 nodes). Moreover, to determine how much of the bacterial diversity is translated to microbe-microbe interactions, we compared the observed features versus the number of nodes (Fig. 8e) or edges (Fig. 8f) found in the microbial co-occurrence networks. We observed that the uninfected, *B. afzelii*, and PBS + *B. afzelii* group with similar number of observed features presented a similar number of interacting nodes in the microbial co-occurrence networks (Fig. 8e). In contrast, for a similar number of observed features, the number of edges increased two times in the *B. afzelii* and PBS + *B. afzelii* group compared to the uninfected one (Fig. 8f). Interestingly, the *E. coli* + *B. afzelii* group with the highest bacterial diversity had also the highest number of interacting nodes and bacterial associations (Fig. 8f). Finally, we tested the robustness of the networks by measuring their tolerance to directed taxa removal and compared it among all the experimental groups. Network inferred from the *E. coli* + *B. afzelii* group presented the lowest values of connectivity loss after directed removal of nodes (Fig. 8g), suggesting the highest tolerance to taxa removal in this network. Statistical comparison of the loss of connectivity for 5 to 7% of nodes removed showed a significant difference between the *E. coli* + *B. afzelii* and the uninfected, *B. afzelii*, or PBS + *B. afzelii* groups (Fig. 8g). All these results showed that significant changes of unique bacterial taxa, high microbial-microbial interactions with unique set of nodes as well as a higher robustness of microbial networks can define the *Borrelia* infection-refractory states in the microbiota of *I. ricinus* fed on immunized mice.

When comparing the microbiota of ticks that received artificially *B. afzelii* or *E. coli* + *B. afzelii* by capillary feeding or microinjection, we found some similarities with the results above in terms of the high number of bacterial taxa (i.e., 470) shared by all the experimental groups (Supplementary Fig. S7a); the high number of unique taxa whose abundance changed significantly in the capillary feeding (i.e., 21) or microinjection (i.e., 31) groups between the *B. afzelii* and *E. coli* + *B. afzelii* (Supplementary Fig. S7b), the clusterization of most of the samples from an experimental condition in separated cluster (Supplementary Fig. S7c). Moreover, when comparing the nodes present in the microbial co-occurrence networks, we found, in contrast to the results above, that the group with the highest number of unique nodes was the group that received *B. afzelii* by capillary feeding (Supplementary Fig. S7d). When we compared the observed features vs the number of nodes or edges, we found that *E. coli* + *B. afzelii* condition from the capillary feeding or microinjection groups had a decreased number of nodes (Supplementary Fig. S7e) and edges (Supplementary Fig. S7f) compared to their control group (*B. afzelii* condition). Finally, we observed a decrease in the robustness of the microbial networks in the *E. coli* + *B. afzelii* condition from the microinjection group compared to the *B. afzelii* group. No significant differences were found between the *E. coli* + *B. afzelii* and *B. afzelii* conditions from the microinjection group (Supplementary Fig. S7g).

These results show that departures from the *B. afzelii*-induced modulation of the microbiota can impact pathogen colonization in *I. ricinus* ticks and that the infection-refractory state is dependent on the techniques used for microbiota perturbation.

## Discussion

Microbiota perturbation is a promising avenue to develop measures to control vector-borne diseases, as vector microbiota influences vector competence [45–47]. Here, we first characterized the interaction of tick microbiota with the pathogen *B. afzelii* and, subsequently, we showed how perturbation of pathogen-induced microbiota led to an infection-refractory state that reduced *Borrelia* colonization in tick vectors.

Other studies found that the presence of *Borrelia* within the vector changed significantly the tick microbiota. For example, *I. ricinus* nymphs molted from larvae that fed on *B. afzelii*-infected mice presented a less abundant but more diverse bacterial microbiome [48]. Differential abundances of several taxa were also found between *B. afzelii*-infected and non-infected ticks [48]. In contrast, a study by Chauhan et al. [49] found no association between the microbiome diversity of a tick and its probability of carrying *B. burgdorferi* but specific microbial taxa were associated with pathogen presence in individual ticks. These results suggest that *Borrelia* infection induced modifications on tick microbiota associated, particularly, to changes in the abundance of bacterial members. Our results also showed that *B. afzelii* produces major shifts in the bacterial community assembly and increases co-occurrence of bacteria, suggesting higher rates of microbe-microbe interactions in infected ticks. Modification of bacterial taxa associations induced by the presence of different pathogens of the genera *Borrelia*, *Anaplasma*, and *Rickettsia* was also previously reported [50]. However, unlike our results, Lejal et al. [50] found that bacterial correlations were lower in the network from *Borrelia*-positive ticks compared to the network from uninfected ticks. These differences could be due to the *Borrelia* species that was considered. In the study of Lejal et al. [50], samples were considered positive for *Borrelia* if it was detected by microfluidic PCR and the bacterial taxa of the same genera was detected by 16S rRNA gene sequencing. Thus, in their study, the *Borrelia* species is unknown. Different species could lead to different results but further comparative studies on the impact of different pathogen species on tick microbiota are needed to affirm this hypothesis. Despite differences found between studies, the evidence shows consistently that the presence of pathogen in the vector modulates the vector microbiota towards a state compatible with pathogen colonization.

Considering that pathogens, such as *Borrelia*, have to overcome several barriers (e.g., evading tick immune defenses or avoiding endocytic digestion in tick gut epithelial cells) to persist in the tick until the next blood meal, they have to activate mechanisms to modulate the gut environment in order to facilitate their colonization and persistence within the vector [3]. Mechanistically, it was shown that *B. burgdorferi* induces the expression of an *I. scapularis* gut gene encoding for PIXR (protein of *I. scapularis* with a reeler domain) [51]. Abrogation of PIXR function in vivo resulted in alterations in the gut microbiome, metabolome, and immune responses and RNA interference-mediated knockdown of PIXR decreased *B. burgdorferi* colonization, which suggest that *B. burgdorferi* induces PIXR expression to enhance colonization in the tick [51]. Similarly, *Anaplasma phagocytophilum*, the agent of human granulocytic anaplasmosis, induces the expression of a gene encoding for *Ixodes scapularis* antifreeze glycoprotein (IAFGP), which perturbs the tick gut microbiota and the integrity of the peritrophic matrix and gut barrier in order to facilitate the infection [52]. The broad effects that the pathogen has on tick gut may create an environment that favors or reduces the fitness of some bacteria, which may explain changes in the diversity, composition, or abundance of taxa in the microbiota. Furthermore, microbiota modulation could lead to the disappearance or emergence of microbial-microbial interactions. In agreement with this, here we found that the keystone taxon *Escherichia-Shigella* [13] was associated to more bacterial taxa in *B. afzelii* network compared to the control network. However, the understanding of the exact role of these newly stablished associations on pathogen persistence within the vector needs further investigation.

Vector microbiota is considered a gate to access vector fitness and competence [53]. Inducing changes in normal tick microbiota can result in pathogen colonization impairment [9]. Here, we showed that tick fed on mice immunized with the Enterobacteriaceae bacteria *E. coli* BL21 presented lower level of *B. afzelii* load within the vector. These results are in concordance with a previous study where mosquitoes fed on immunized birds against two strains of *E. coli*, namely O86:B7 and BL21, had reduced number of *P. relictum* (causal agent of avian malaria) oocysts in the midguts [15]. Reduction of *Plasmodium* infectivity was owed to the alteration of *Plasmodium*-induced modulation of the mosquito microbiota [15]. Similarly, in the present study, we found that anti-microbiota vaccine produced changes in the microbial community assembly different from those induced by *B. afzelii*. Interestingly, we also found that anti-microbiota vaccine reduced the relative importance of the taxon *Escherichia-Shigella* compared to the same taxon in PBS + *B. afzelii* group. These results suggest that targeting Enterobacteriaceae with host antibodies induce the modulation of the vector microbiota triggering a cascading ecological impact on the whole tick microbiota that resulted in the impairment of pathogen colonization. Supporting this idea, Narasimhan et al. [9] reported that an experimentally induced dysbiosed microbiota due to tick rearing in a sterile environment reduced *B. burgdorferi s.s.* colonization in tick midgut. It was also reported that this outcome was associated with a lower expression of the transcription factor STAT and the glycoprotein peritrophin that affected the integrity of the peritrophic matrix, which is essential for *B. burgdorferi s.s.* successful colonization. In addition, Rana et al. [54] demonstrated that IFNγ, present in *Borrelia*-infected mice, can activate the Dome1-JAK-STAT pathway, which could impact tick microbiota, pathogen colonization, and transmission to a naïve host. The simultaneous acquisition of host factors (e.g., IFNγ) and anti-microbiota antibodies induced by immunization in the context of *Borrelia* infection could explain some of the difference we observed between the microbiota of tick fed on infected mice as opposed to tick fed on capillary or microinjected. Whether these mechanisms are implicated in the effects of anti-microbiota vaccine remains to be elucidated.

Enterobacteriaceae was suggested to have a role in *B. burgdorferi* colonization since its abundance was negatively correlated with the pathogen abundance [55]. Here, we found that antibodies-mediated targeting of Enterobacteriaceae modulate the tick microbiota and reduced *B. afzelii* load within the tick. These results suggest that the commensal bacteria Enterobacteriaceae may have a key role on pathogen acquisition. However, when we experimentally introduced *E. coli* BL21 with *B. afzelii* to the midgut of *I. ricinus* nymphs by capillary feeding or microinjection, we found surprisingly that *B. afzelii* level on these nymphs were lower compared to nymphs that only received *B. afzelii.* These results suggest that the observed reduction on the pathogen load is not due to the shift in the abundance of Enterobacteriaceae. It is rather likely due to the modulation of the microbiota (by the depletion or emergence of some bacteria) towards a different state from the one induced by *B. afzelii*, specifically, a *Borrelia*-refractory state incompatible with pathogen development. It has been shown that alteration of the vertebrate gut microbiota composition either by antibiotic treatment [9] or by addition of probiotics [39] can inhibit the colonization of pathogenic bacteria in the gut. When we defined this infection-refractory state by comparing taxonomic profile, abundance, and emerging properties of tick microbiota, we found that the main differences between the uninfected, the pathogen-permissive, and the infection-refractory state relied mostly in the fluctuation of the abundance of unique taxa and the emergent properties of microbial networks. Emergent properties are one of the strengths of biological networks and they can help explaining the behavior of complex systems [56, 57]. We found that changes induced by *B. afzelii* in terms of interacting nodes, microbial-microbial interactions, and robustness of the microbial network were not different as much as those induced by *E. coli* + *B. afzelii* compared to the uninfected network when ticks are exposed to immunized mice. Notably, a large-edge number, a higher number of new interconnecting taxa, and a high robustness were found in the *E. coli* + *B. afzelii* network compared to the uninfected or *B. afzelii* networks. When a commensal bacterium was added artificially, we found in contrast a lower number of edges and interconnecting taxa as well as a lower robustness. These results suggest that perturbation of the microbiota by different means can lead to different infection-refractory states. Similar results were found in tick microbiota after disturbance with an anti-tick vaccine, *A. phagocytophilum* infection and antimicrobial peptide, where a higher number of associations, but lower robustness, were found [58]. These results suggest that tick microbiota is highly susceptible to perturbations that led to changes in the emergent properties of microbial networks. Specifically, here, we found that perturbation of the microbiota with anti-microbiota vaccine induced drastic changes in the bacterial community assembly that led to an unsuitable stage for *B. afzelii* colonization within the vector. It is noteworthy that, recently, Narasimhan et al. [59] demonstrated that perturbation of the microbiome composition by different strategies (raise of *I. scapularis* in germ-free isolators for the generation of larvae harboring no environmental bacteria, feeding gentamicin-resistant *B. burgdorferi*-infected ticks on gentamicin-injected mice, RNAi-mediated knockdown of *stat*) does not influence *B. burgdorferi* burden on *I. scapularis* ticks. Interestingly, none of the methods for perturbation of tick microbiota used by us in the present study (anti-microbiota vaccine and addition of the commensal bacteria *E. coli*) were included in the study of Narasimhan et al. [59]. This suggests that the technique used for the perturbation of the microbiota may influence the outcome on pathogen colonization and highlights the potential of the use of anti-microbiota vaccine for the control of *Borrelia* colonization in the ticks.

Finally, we found that ticks fed on mice immunized with *E. coli* BL21 and infected with *B. afzelii* had significantly lower mortality rates compared with the PBS + *B. afzelii* group. This result is in contrast with one study where they did not find evidence that *B. afzelii* infection and reduction of larval microbiota (by egg surface sterilization with bleach and ethanol) impacted tick survival [60]. Differences could be explained by the different developmental stages where the measure was done. In our study, tick mortality was measured in engorged larvae while in the study of Hurry et al. [60], survival rates were measured in nymphs. We hypothesized that the diminution of the mortality of *E. coli* + *B. afzelii* larvae could be due to the lower *B. afzelii* load within the ticks, which could favor to the fitness of the vector.

## Conclusions

We found that *B. afzelii* infection modulates the *I. ricinus* microbiota in terms of beta diversity, composition, abundance, and microbial co-occurrence. The broad effects induced by the pathogen on tick microbiota are likely the result of the pathogen generating an environment conducive for its colonization within the vector. Disrupting this infection-permissive microbiome state may be an alternative to block pathogen colonization and its subsequent transmission to a new host. Effective chains of infection of vector-borne pathogens involve a competent vector, an infective pathogen, and an infection-permissive microbiome [61], and mismatch of one of these components can result in the inability of the pathogen to efficiently colonize the vector gut and/or the inability of the vector to transmit pathogens [61]. Here, we showed that anti-microbiota vaccine targeting *Escherichia-Shigella* can shape *I. ricinus* microbiome towards an infection-refractory state, by shifting the abundance of several bacterial members of the microbiota, and increasing microbe-microbe interactions and robustness, which impacted the whole tick microbiota and resulted in a lower *B. afzelii* load within the vector. Therefore, anti-microbiota vaccine is a suitable tool for the manipulation of the microbiome towards a desired state and can be used for the control of vector-borne diseases.

## Supplementary Information

**Additional file 1:** **Supplementary Fig. S1.** Changes in the taxonomic profile of tick microbiota after *B. afzelii* infection. Volcano plot showing the differential microbial abundance in tick microbiota from the uninfected and *B. afzelii* groups. Turquoise and purple dots represent bacterial taxa whose abundances significantly decreased and increased, respectively, in the microbiota of ticks from *B. afzelii* group compared to the control group. Heatmaps represent the abundance (expressed as CLR) of all the taxa with significant differences between the uninfected and *B. afzelii* groups. Taxa whose abundance decreased significantly in the *B. afzelii* group are annotated in turquoise. Taxonomic table used for the differential abundance analysis were obtained from 16S rRNA gene sequences from ticks fed on uninfected mice (*n* = 10 individual larvae) and *B. afzelii*-infected mice (*n* = 10 individual larvae).

**Additional file 2:** **Supplementary Fig. S2. **Detection of *Borrelia* proteins*. *Proteins of *Borrelia* were detected by western blot using sera of mice experimentally infected with *B. afzelii* and immunized with a live vaccine containing *E. coli* BL21 or a mock vaccine (PBS).

**Additional file 3:** **Supplementary Fig. S3.** Changes in the taxonomic profile of tick microbiota after *B. afzelii* infection and anti-microbiota vaccine immunization. Volcano plot showing the differential microbial abundance in tick microbiota from the PBS+*B. afzelii* and *E. coli*+*B. afzelii* groups. Green and pink dots represent bacterial taxa whose abundances significantly decreased and increased, respectively, in the microbiota of ticks from *E. coli*+*B. afzelii* group compared to the PBS+*B. afzelii* group. Heatmaps represent the abundance (expressed as CLR) of all the taxa with significant differences between the PBS+*B. afzelii* and *E. coli*+*B. afzelii* groups. Taxa whose abundance decreased significantly in the *E. coli*+*B. afzelii* group are annotated in green. Taxonomic table used for the differential abundance analysis were obtained from 16S rRNA gene sequences from ticks fed on PBS+*B. afzelii* mice (*n* = 10 individual larva) and *E. coli*+*B. afzelii* mice (*n* = 8 individual larva).

**Additional file 4:** **Supplementary Fig. S4. **Impact of the addition of a commensal bacterium in the alpha and beta diversity of tick microbiota. (a) Faith’s phylogenetic diversity and (b) Pielou’s evenness indexes were used to measure the richness and evenness, respectively, of microbiota of ticks that received *B. afzelii* or *E. coli+B. afzelii* by capillary feeding or anal pore injection (Kruskal-Wallis, *p < 0.05*). Beta diversity of tick microbiota were analyzed with the (c) Jaccard and (d) Weighted Unifrac indexes to measure the similarity between the bacterial communities in the microbiota of ticks that received *B. afzelii* *or E. coli+B. afzelii* by capillary feeding or anal pore injection different experimental conditions (PERMANOVA, *p < 0.05*).

**Additional file 5:** **Supplementary Fig. S5. **Detection of Enterobacteriaceae by PCR. Representative images of the gel of electrophoresis showing bands corresponding to the 16S rRNA gene for Enterobacteriaceae. Different panels represent different experiments: Ticks were given *B. afzelii* or *E. coli*+*B. afzelii* by capillary feeding and incubated for 6h after the feeding (upper panel) or by microinjection and incubated for 6h after the injection (lower panel). Each lane represents a different tick from groups. For the positive control was used DNA extracted from a culture of *E. coli* BL21.

**Additional file 6:** **Supplementary Fig. S6. **Cluster analysis of different samples of tick microbiota. Dendrogram based on Ward’s method of clustering for samples of tick microbiota from the uninfected, *B. afzelii*, PBS+*B. afzelii* and *E. coli*+*B. afzelii* groups.

**Additional file 7:** **Supplementary Fig. S7. **Impact of the addition of a commensal bacterium on the emergent properties of I*. ricinus* microbiota. Venn diagram showing (a) the common and unique bacterial taxa among the tick microbiota that received *B. afzelii* or *E. coli+B. afzelii* by capillary feeding and anal pore injection (b) the shared and unique taxa whose abundance changed significantly between *B. afzelii* vs. *E. coli*+*B. afzelii* comparisons in capillary feeding and anal pore injection groups, (c) Dendrogram of clustering for samples of tick microbiota from different experimental conditions, (d) Venn diagram showing the common and unique nodes found in microbial co-occurrence networks from all conditions. Scatter plot showing the mean of observed features versus number of (e) nodes and (f) edges found in the microbial co-occurrence networks and (g) Scatter plot showing the loss of connectivity when 5 to 7% of nodes are removed from the microbial co-occurrence network.

**Additional file 8:** **Supplementary Table S1.** Bacterial taxa found as contaminants in the 16S rRNA gene sequencing datasets from ticks fed on mice in different experimental conditions. Contaminants were statistically identified (TRUE) and removed from the 16S rRNA gene sequencing datasets using the decontam R package

**Additional file 9:** **Supplementary Table S2. **Jaccard indexes of local centrality measures for the comparison between uninfected and *B. afzelii* groups. Jaccard’s indexes for each of local centrality measures (i.e., degree, betweenness centrality, closeness centrality, eigenvector centrality and hub taxa) of the sets of most central nodes for pairwise network comparisons. The two p-values, P(J ≤ j) and P(J ≥ j), for each Jaccard’s index were added.

**Additional file 10:** **Supplementary Table S3. **Common and unique neighbor nodes of the taxon *Escherichia-Shigella* between the uninfected and *B. afzelii* groups.

**Additional file 11:** **Supplementary Table S4. **Mice tissues positive for *B. afzelii* in PBS+*B. afzelii* and E*. coli+B. afzelii. *Heart, skin and right ankle joint were tested for *B. afzelii* DNA and positive tissues were listed in the panel a and b for PBS+*B. afzelii* and E*. coli+B. afzelii, *respectively*. *Heart and skin were tested for* B. afzelii *RNA and positive tissues were listed in the panel c and d for PBS+*B. afzelii* and E*. coli+B. afzelii, *respectively.

**Additional file 12:** **Supplementary Table S5. **Jaccard indexes of local centrality measures for the comparison between PBS+*B. afzelii* vs. *E. coli+B. afzelii* groups. Jaccard’s indexes for each of local centrality measures (i.e., degree, betweenness centrality, closeness centrality, eigenvector centrality and hub taxa) of the sets of most central nodes for pairwise network comparisons. The two p-values, P(J ≤ j) and P(J ≥ j), for each Jaccard’s index were added

**Additional file 13:** **Supplementary Table S6. **Common and unique neighbor nodes of the taxon *Escherichia-Shigella* between the PBS+*B. afzelii *and *E. coli+B. afzelii* groups.

**Additional file 14:** **Supplementary Table S7.** Bacterial taxa found as contaminants in the 16S rRNA gene sequencing datasets from ticks exposed to *B. afzelii* or *E. coli+B. afzelii* by capillary feeding or anal pore microinjection. Contaminants were statistically identified (TRUE) and removed from the 16S rRNA gene sequencing datasets using the decontam R package.

## Data Availability

The datasets presented in this study can be found in online repositories. The names of the repository/repositories and accession number(s) can be found below: https://www.ncbi.nlm.nih.gov/sra, PRJNA870490.

## References

[1] Madison-Antenucci S, Kramer LD, Gebhardt LL, Kauffman E (2020). Emerging tick-borne diseases. Clin Microbiol Rev.

[2] Radolf JD, Caimano MJ, Stevenson B, Hu LT (2012). Of ticks, mice and men: understanding the dual-host lifestyle of Lyme disease spirochaetes. Nat Rev Microbiol.

[3] Kurokawa C, Lynn GE, Pedra JHF, Pal U, Narasimhan S, Fikrig E (2020). Interactions between *Borrelia burgdorferi* and ticks. Nat Rev Microbiol.

[4] Barbour AG, Fish D (1993). The biological and social phenomenon of Lyme disease. Science.

[5] Richter D, Klug B, Spielman A, Matuschka FR (2004). Adaptation of diverse Lyme disease spirochetes in a natural rodent reservoir host. Infect Immun.

[6] Cirimotich CM, Ramirez JL, Dimopoulos G (2011). Native microbiota shape insect vector competence for human pathogens. Cell Host Microbe.

[7] Dong Y, Manfredini F, Dimopoulos G (2009). Implication of the mosquito midgut microbiota in the defense against malaria parasites. PLoS Pathog.

[8] Xi Z, Ramirez JL, Dimopoulos G (2008). The *Aedes aegypti* Toll pathway controls Dengue virus infection. PLoS Pathog.

[9] Narasimhan S, Rajeevan N, Liu L, Zhao YO, Heisig J, Pan J (2014). Gut microbiota of the tick vector *Ixodes scapularis* modulate colonization of the Lyme disease spirochete. Cell Host Microbe.

[10] Bando H, Okado K, Guelbeogo WM, Badolo A, Aonuma H, Nelson B (2013). Intra-specific diversity of *Serratia marcescens* in *Anopheles* mosquito midgut defines *Plasmodium* transmission capacity. Sci Rep.

[11] Gall CA, Reif KE, Scoles GA, Mason KL, Mousel M, Noh SM (2016). The bacterial microbiome of *Dermacentor andersoni* ticks influences pathogen susceptibility. ISME J.

[12] Landesman WJ, Mulder K, Fredericks LP, Allan BF. Cross-kingdom analysis of nymphal-stage Ixodes scapularis microbial communities in relation to Borrelia burgdorferi infection and load. FEMS Microbiol Ecol. 2019;95(12):fiz167. 10.1093/femsec/fiz167. 10.1093/femsec/fiz167 PMC6859517 31722384

[13] Mateos-Hernández L, Obregón D, Maye J, Bornères J, Versille N, de la Fuente J (2020). Anti-tick microbiota vaccine impacts *Ixodes ricinus* performance during feeding. Vaccines (Basel).

[14] Mateos-Hernández L, Obregón D, Wu-Chuang A, Maye J, Bornères J, Versillé N (2021). Anti-microbiota vaccines modulate the tick microbiome in a taxon-specific manner. Front Immunol.

[15] Aželytė J, Wu-Chuang A, Žiegytė R, Platonova E, Mateos-Hernandez L, Maye J (2022). Anti-microbiota vaccine reduces avian malaria infection within mosquito vectors. Front Immunol.

[16] Schwan TG, Simpson WJ, Rosa PA (1991). Laboratory confirmation of Lyme disease. Can J Infect Dis.

[17] Mateos-Hernández L, Rakotobe S, Defaye B, Cabezas-Cruz A, Šimo L (2020). A capsule-based model for immature hard tick stages infestation on laboratory mice. J Vis Exp.

[18] Castillo M, Martín-Orúe SM, Manzanilla EG, Badiola I, Martín M, Gasa J (2006). Quantification of total bacteria, enterobacteria and lactobacilli populations in pig digesta by real-time PCR. Vet Microbiol.

[19] Davis NM, Proctor DM, Holmes SP, Relman DA, Callahan BJ (2018). Simple statistical identification and removal of contaminant sequences in marker-gene and metagenomics data. Microbiome.

[20] Bolyen E, Rideout JR, Dillon MR, Bokulich NA, Abnet M, Asnicar F (2019). Reproducible, interactive, scalable and extensible microbiome data science using QIIME 2. Nat Biotechnol.

[21] Callahan BJ, McMurdie PJ, Rosen MJ, Han AW, Johnson AJA, Holmes SP (2016). DADA2: High-resolution sample inference from Illumina amplicon data. Nat Methods.

[22] Bokulich NA, Kaehler BD, Rideout JR, Dillon M, Bolyen E, Knight R (2018). Optimizing taxonomic classification of marker-gene amplicon sequences with QIIME 2’s q2-feature-classifier plugin. Microbiome.

[23] Yarza P, Yilmaz P, Pruesse E, Glöckner FO, Ludwig W, Schleifer KH (2014). Uniting the classification of cultured and uncultured bacteria and archaea using 16S rRNA gene sequences. Nat Rev Microbiol.

[24] Margos G, Gofton A, Wibberg D, Dangel A, Marosevic D, Loh SM (2018). The genus Borrelia reloaded. PLoS ONE.

[25] Adeolu  M, Gupta RS (2014). A phylogenomic and molecular marker-based proposal for the division of the genus *Borrelia* into two genera: the emended genus *Borrelia* containing only the members of the relapsing fever *Borrelia*, and the genus *Borreliella* gen. nov. containing the members of the Lyme disease Borrelia (*Borrelia burgdorferi* sensu lato complex). Antonie van Leeuwenhoek, Int J Gen Mol Microbiol.

[26] Gupta RS. Distinction between Borrelia and Borreliella is more robustly supported by molecular and phenotypic characteristics than all other neighbouring prokaryotic genera: Response to Margos' et al. "The genus Borrelia reloaded" (PLoS ONE 13(12): e0208432). PLoS One. 2019;14(8):e0221397. 10.1371/journal.pone.0221397. 10.1371/journal.pone.0221397 PMC6711536 31454394

[27] Friedman J, Alm EJ (2012). Inferring correlation networks from genomic survey data. PLoS Comput Biol.

[28] RStudio Team. RStudio: Integrated Development for R. Boston: RStudio, PBC; 2020. http://www.rstudio.com/.

[29] Bastian M, Heymann S, Jacomy, M. Gephi: An Open Source Software for Exploring and Manipulating Networks. Proceedings of the International AAAI Conference on Web and Social Media. 2009;3(1):361–62. 10.1609/icwsm.v3i1.13937.

[30] Lhomme S. NetSwan: Network Strengths and Weaknesses Analysis. R Pack Version. 2015. https://rdrr.io/cran/NetSwan/. Accessed Sept 2022.

[31] Peschel S, Müller CL, von Mutius E, Boulesteix AL, Depner M. NetCoMi: network construction and comparison for microbiome data in R. Brief Bioinform. 2021;22(4):bbaa290. 10.1093/bib/bbaa290. 10.1093/bib/bbaa290 PMC8293835 33264391

[32] Fernandes AD, Reid JN, Macklaim JM, McMurrough TA, Edgell DR, Gloor GB (2014). Unifying the analysis of high-throughput sequencing datasets: characterizing RNA-seq, 16S rRNA gene sequencing and selective growth experiments by compositional data analysis. Microbiome.

[33] Love MI, Huber W, Anders S (2014). Moderated estimation of fold change and dispersion for RNA-seq data with DESeq2. Genome Biol.

[34] Oksanen J, Simpson GL, Blanchet FG, Kindt R, Legendre P, Minchin PR, et al. Vegan: Community Ecology Package. R Packag version 26-0. 2021. https://CRAN.R-project.org/package=vegan. Accessed Aug 2023.

[35] Fawcett PT, Rose CD, Gibney KM (1995). Comparative evaluation of adsorption with *E. coli* on ELISA tests for Lyme borreliosis. J Rheumatol..

[36] Bruckbauer HR, Preac-Mursic V, Fuchs R, Wilske B (1992). Cross-reactive proteins of *Borrelia burgdorferi*. Eur J Clin Microbiol Infect Dis.

[37] Liu Y, Wang J, Wu C (2022). Modulation of gut microbiota and immune system by probiotics, pre-biotics, and post-biotics. Front Nutr.

[38] Everard A, Matamoros S, Geurts L, Delzenne NM, Cani PD (2014). *Saccharomyces boulardii* administration changes gut microbiota and reduces hepatic steatosis, low-grade inflammation, and fat mass in obese and type 2 diabetic db/db mice. MBio.

[39] Wang X, Zhang P, Zhang X (2021). Probiotics regulate gut microbiota: an effective method to improve immunity. Molecules.

[40] Tuo Y, Song X, Song Y, Liu W, Tang Y, Gao Y (2018). Screening probiotics from *Lactobacillus* strains according to their abilities to inhibit pathogen adhesion and induction of pro-inflammatory cytokine IL-8. J Dairy Sci.

[41] Surendran Nair M, Amalaradjou MA, Venkitanarayanan K (2017). Antivirulence properties of probiotics in combating microbial pathogenesis. Adv Appl Microbiol.

[42] Fang K, Jin X, Hong SH. Probiotic Escherichia coli inhibits biofilm formation of pathogenic *E. coli* via extracellular activity of DegP. Sci Rep. 2018;8(1):4939. 10.1038/s41598-018-23180-1. 10.1038/s41598-018-23180-1 PMC5862908 29563542

[43] Studier FW, Moffatt BA (1986). Use of bacteriophage T7 RNA polymerase to direct selective high-level expression of cloned genes. J Mol Biol.

[44] Jeong H, Kim HJ, Lee SJ (2015). Complete genome sequence of *Escherichia coli* strain BL21. Genome Announc.

[45] Narasimhan S, Swei A, Abouneameh S, Pal U, Pedra JHF, Fikrig E (2021). Grappling with the tick microbiome. Trends Parasitol.

[46] Wu-Chuang A, Hodžić A, Mateos-Hernández L, Estrada-Peña A, Obregon D, Cabezas-Cruz A (2021). Current debates and advances in tick microbiome research. Curr Res Parasitol Vector-Borne Dis.

[47] Dennison NJ, Jupatanakul N, Dimopoulos G (2014). The mosquito microbiota influences vector competence for human pathogens. Curr Opin Insect Sci.

[48] Hamilton PT, Maluenda E, Sarr A, Belli A, Hurry G, Duron O (2021). *Borrelia afzelii* infection in the rodent host has dramatic effects on the bacterial microbiome of *Ixodes ricinus* ticks. Appl Environ Microbiol.

[49] Chauhan G, McClure J, Hekman J, Marsh PW, Bailey JA, Daniels RF (2020). Combining citizen science and genomics to investigate tick, pathogen, and commensal microbiome at single-tick resolution. Front Genet.

[50] Lejal E, Chiquet J, Aubert J, Robin S, Estrada-Peña A, Rue O (2021). Temporal patterns in *Ixodes ricinus* microbial communities: an insight into tick-borne microbe interactions. Microbiome.

[51] Narasimhan S, Schuijt TJ, Abraham NM, Rajeevan N, Coumou J, Graham M (2017). Modulation of the tick gut milieu by a secreted tick protein favors *Borrelia burgdorferi* colonization. Nat Commun.

[52] Abraham NM, Liu L, Jutras BL, Yadav AK, Narasimhan S, Gopalakrishnan V (2017). Pathogen-mediated manipulation of arthropod microbiota to promote infection. Proc Natl Acad Sci U S A.

[53] Wu-Chuang A, Obregon D, Mateos-Hernández L, Cabezas-Cruz A (2022). Anti-tick microbiota vaccines: how can this actually work?. Biologia.

[54] Rana VS , Kitsou C, Dutta S, Ronzetti MH, Zhang M, Bernard Q (2023). Dome1-JAK-STAT signaling between parasite and host integrates vector immunity and development. Science.

[55] Ross BD, Hayes B, Radey MC, Lee X, Josek T, Bjork J (2018). *Ixodes scapularis* does not harbor a stable midgut microbiome. ISME J.

[56] Aderem A (2005). Systems biology: Its practice and challenges. Cell.

[57] Röttjers L, Faust K (2018). From hairballs to hypotheses–biological insights from microbial networks. FEMS Microbiol Rev.

[58] Estrada-Peña A, Cabezas-Cruz A, Obregón D (2020). Resistance of tick gut microbiome to anti-tick vaccines, pathogen infection and antimicrobial peptides. Pathogens.

[59] Narasimhan S, Rajeevan N, Graham M, Wu MJ, DePonte K, Marion S (2022). Tick transmission of *Borrelia burgdorferi* to the murine host is not influenced by environmentally acquired midgut microbiota. Microbiome.

[60] Hurry G, Maluenda E, Sarr A, Belli A, Hamilton PT, Duron O (2021). Infection with *Borrelia afzelii* and manipulation of the egg surface microbiota have no effect on the fitness of immature *Ixodes ricinus* ticks. Sci Reports.

[61] Maitre A, Wu-Chuang A, Aželytė J, Palinauskas V, Mateos-Hernandez L, Obregon D (2022). Vector microbiota manipulation by host antibodies: the forgotten strategy to develop transmission-blocking vaccines. Parasit Vectors.

