# Supplementary figures and images for: Microbiota perturbation by anti-microbiota vaccine reduces the colonization of Borrelia afzelii in Ixodes ricinus
Source: Microbiome. 2023 Jul 24;11:151. doi: 10.1186/s40168-023-01599-7 (PMC10364381; doi:10.1186/s40168-023-01599-7)

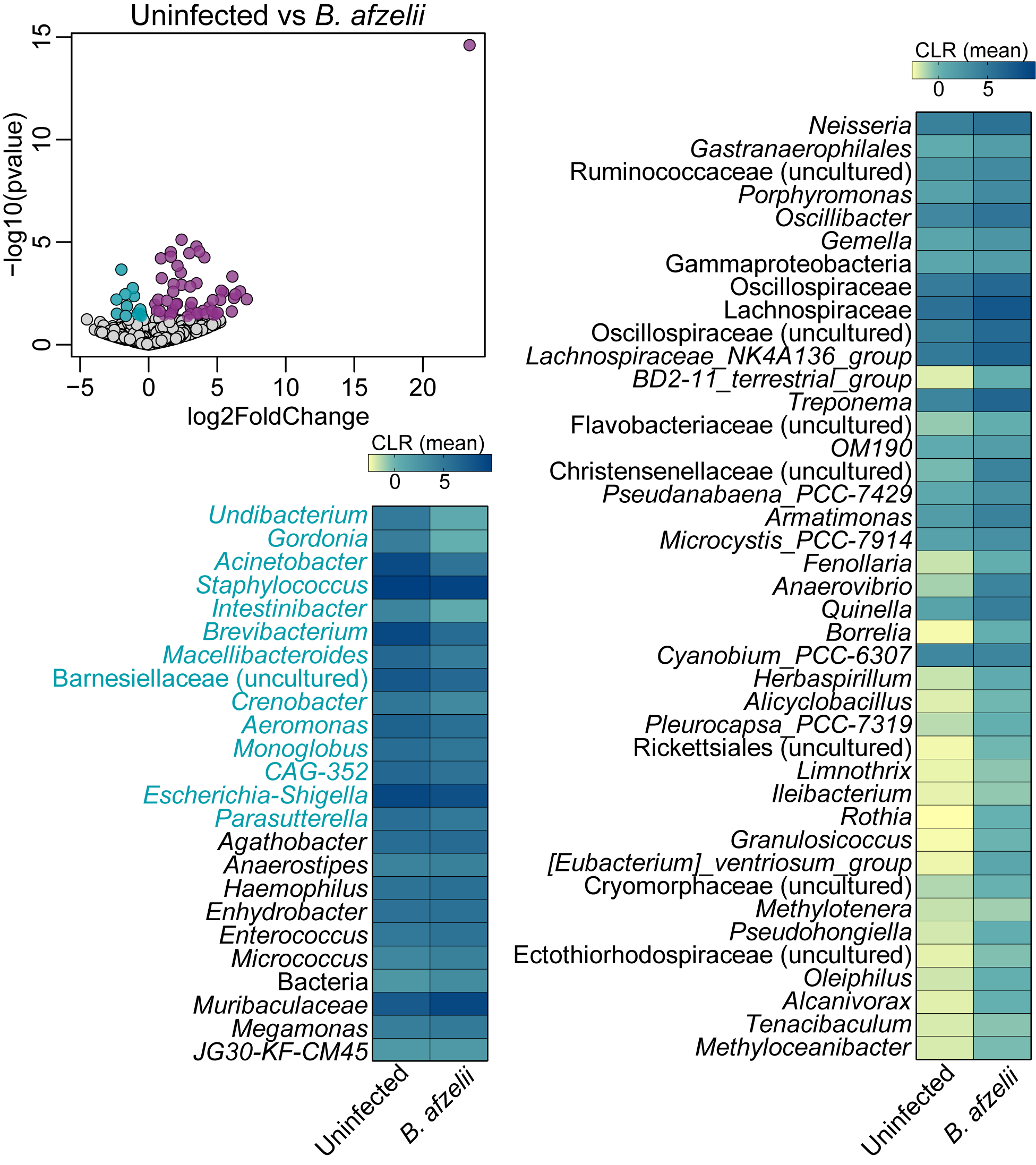

Supplement: Supplementary file 2 — Additional file 1: Supplementary Fig. S1. Changes in the taxonomic profile of tick microbiota after B. afzelii infection. Volcano plot showing the differential microbial abundance in tick microbiota from the uninfected and B. afzelii groups. Turquoise and purple dots represent bacterial taxa whose abundances significantly decreased and increased, respectively, in the microbiota of ticks from B. afzelii group compared to the control group. Heatmaps represent the abundance (expressed as CLR) of all the taxa with significant differences between the uninfected and B. afzelii groups. Taxa whose abundance decreased significantly in the B. afzelii group are annotated in turquoise. Taxonomic table used for the differential abundance analysis were obtained from 16S rRNA gene sequences from ticks fed on uninfected mice (n = 10 individual larvae) and B. afzelii-infected mice (n = 10 individual larvae). [file 40168_2023_1599_MOESM1_ESM.tif]

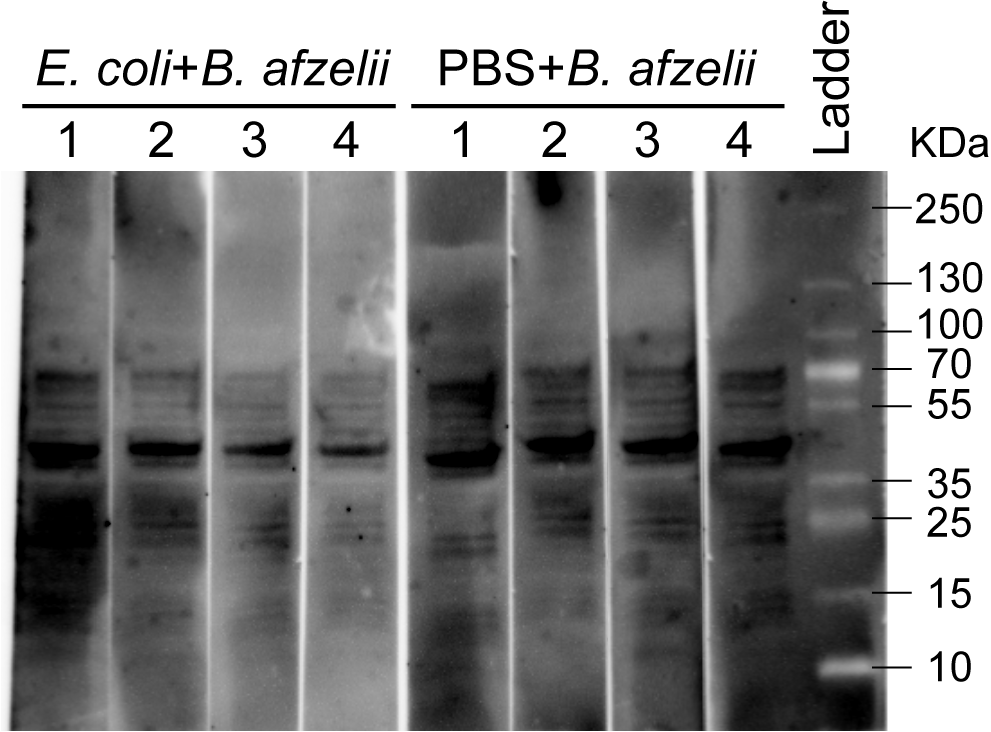

Supplement: Supplementary file 3 — Additional file 2: Supplementary Fig. S2. Detection of Borrelia proteins. Proteins of Borrelia were detected by western blot using sera of mice experimentally infected with B. afzelii and immunized with a live vaccine containing E. coli BL21 or a mock vaccine (PBS). [file 40168_2023_1599_MOESM2_ESM.tif]

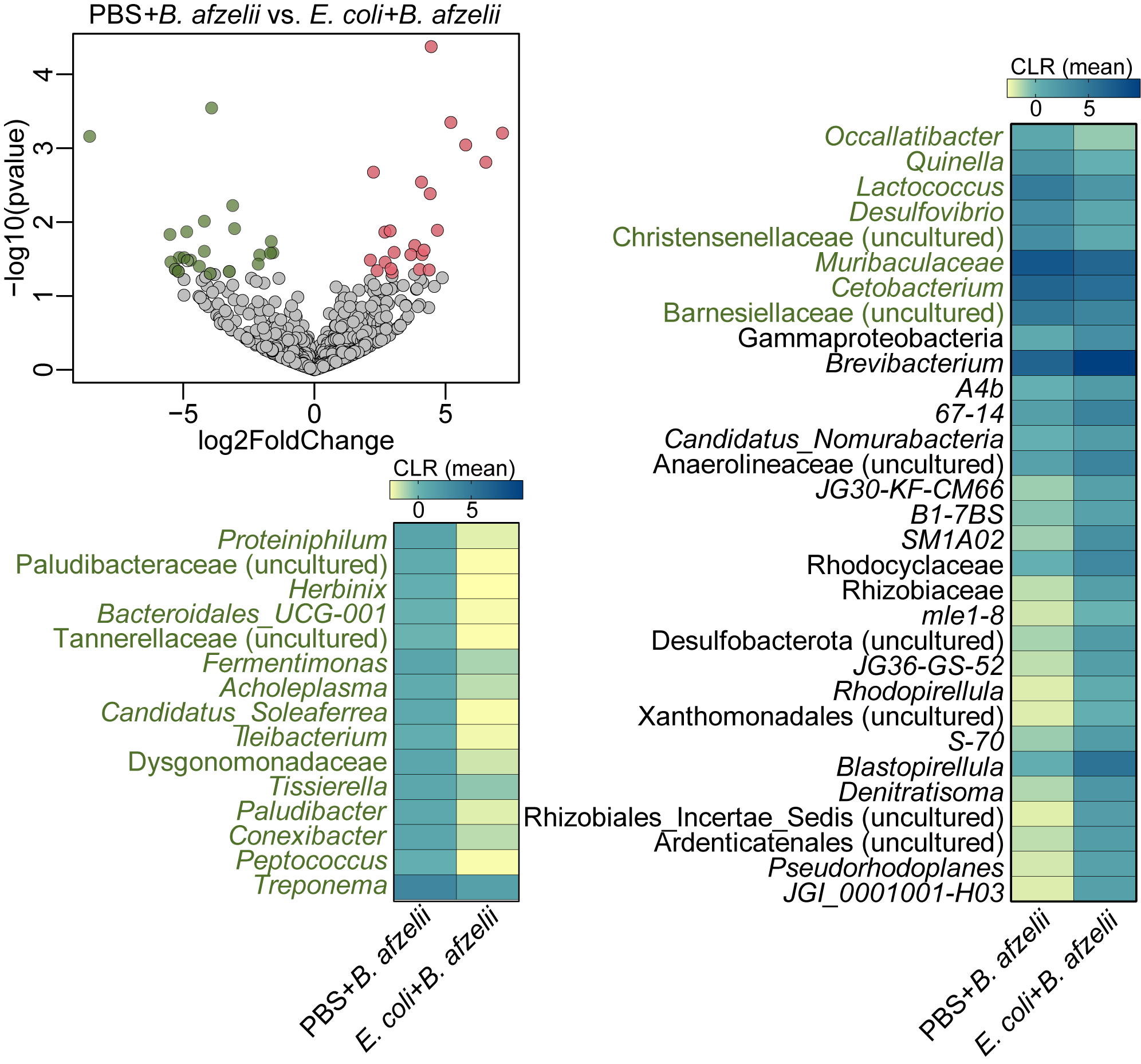

Supplement: Supplementary file 4 — Additional file 3: Supplementary Fig. S3. Changes in the taxonomic profile of tick microbiota after B. afzelii infection and anti-microbiota vaccine immunization. Volcano plot showing the differential microbial abundance in tick microbiota from the PBS+B. afzelii and E. coli+B. afzelii groups. Green and pink dots represent bacterial taxa whose abundances significantly decreased and increased, respectively, in the microbiota of ticks from E. coli+B. afzelii group compared to the PBS+B. afzelii group. Heatmaps represent the abundance (expressed as CLR) of all the taxa with significant differences between the PBS+B. afzelii and E. coli+B. afzelii groups. Taxa whose abundance decreased significantly in the E. coli+B. afzelii group are annotated in green. Taxonomic table used for the differential abundance analysis were obtained from 16S rRNA gene sequences from ticks fed on PBS+B. afzelii mice (n = 10 individual larva) and E. coli+B. afzelii mice (n = 8 individual larva). [file 40168_2023_1599_MOESM3_ESM.tif]

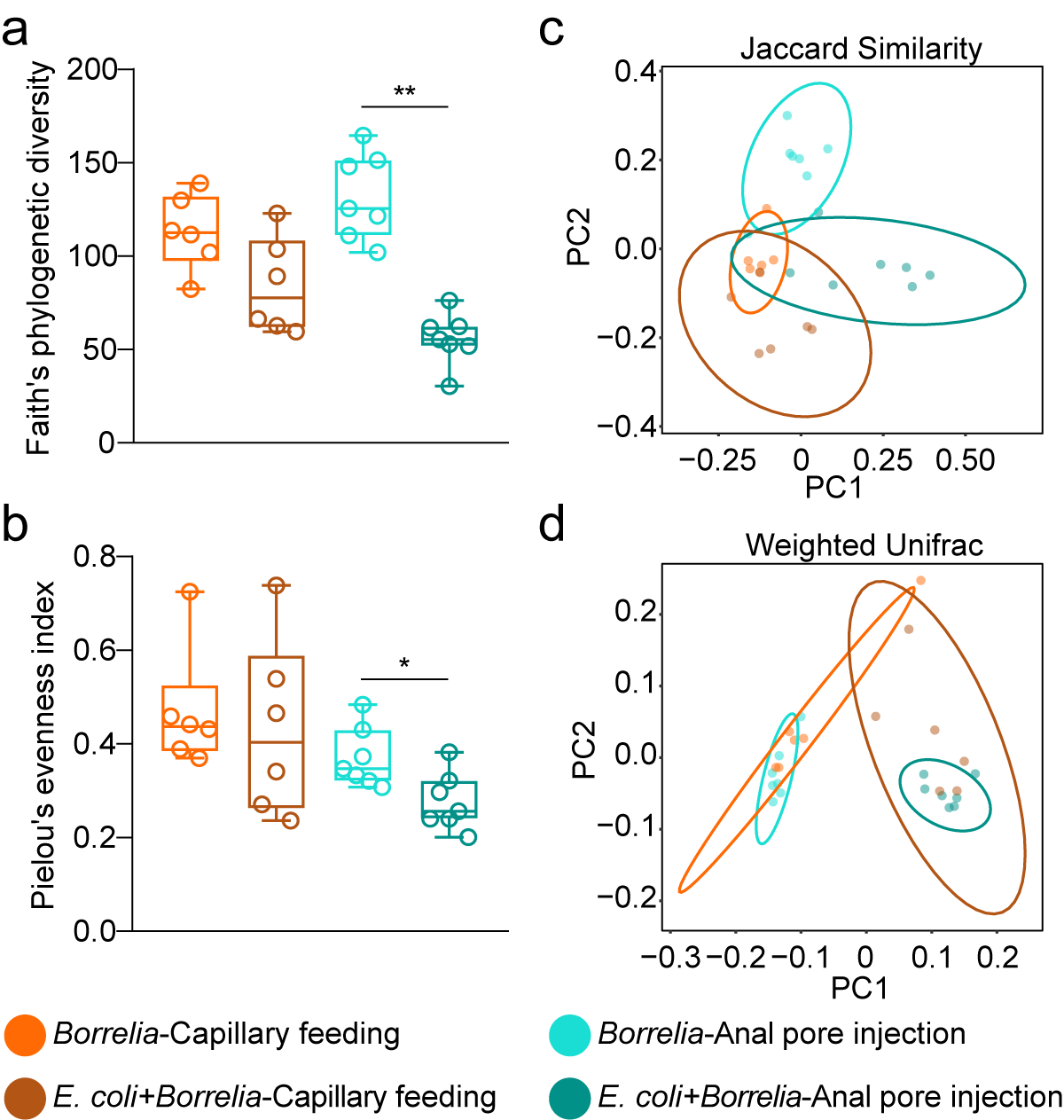

Supplement: Supplementary file 5 — Additional file 4: Supplementary Fig. S4. Impact of the addition of a commensal bacterium in the alpha and beta diversity of tick microbiota. (a) Faith’s phylogenetic diversity and (b) Pielou’s evenness indexes were used to measure the richness and evenness, respectively, of microbiota of ticks that received B. afzelii or E. coli+B. afzelii by capillary feeding or anal pore injection (Kruskal-Wallis, p < 0.05). Beta diversity of tick microbiota were analyzed with the (c) Jaccard and (d) Weighted Unifrac indexes to measure the similarity between the bacterial communities in the microbiota of ticks that received B. afzelii or E. coli+B. afzelii by capillary feeding or anal pore injection different experimental conditions (PERMANOVA, p < 0.05). [file 40168_2023_1599_MOESM4_ESM.tif]

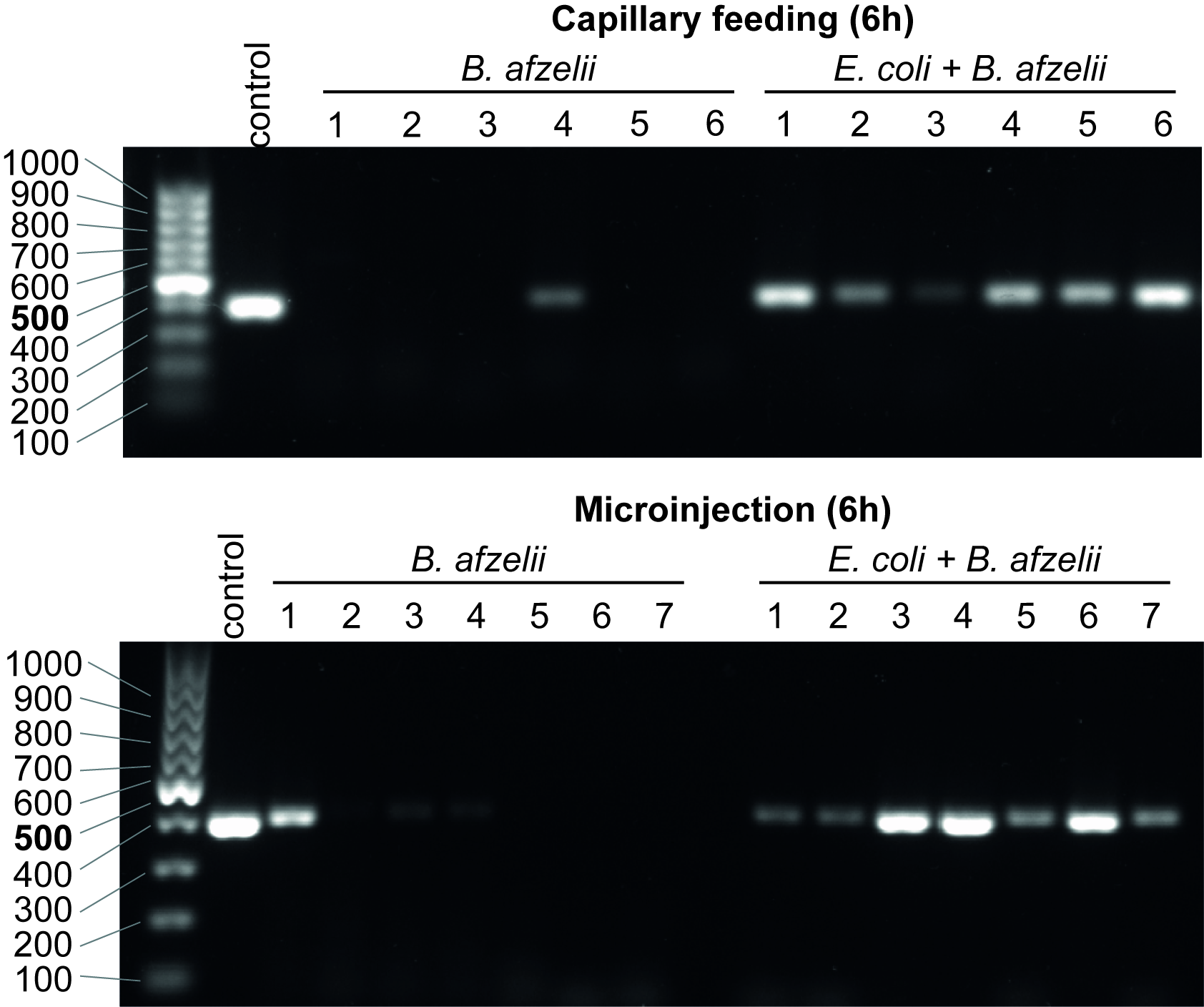

Supplement: Supplementary file 6 — Additional file 5: Supplementary Fig. S5. Detection of Enterobacteriaceae by PCR. Representative images of the gel of electrophoresis showing bands corresponding to the 16S rRNA gene for Enterobacteriaceae. Different panels represent different experiments: Ticks were given B. afzelii or E. coli+B. afzelii by capillary feeding and incubated for 6h after the feeding (upper panel) or by microinjection and incubated for 6h after the injection (lower panel). Each lane represents a different tick from groups. For the positive control was used DNA extracted from a culture of E. coli BL21. [file 40168_2023_1599_MOESM5_ESM.tif]

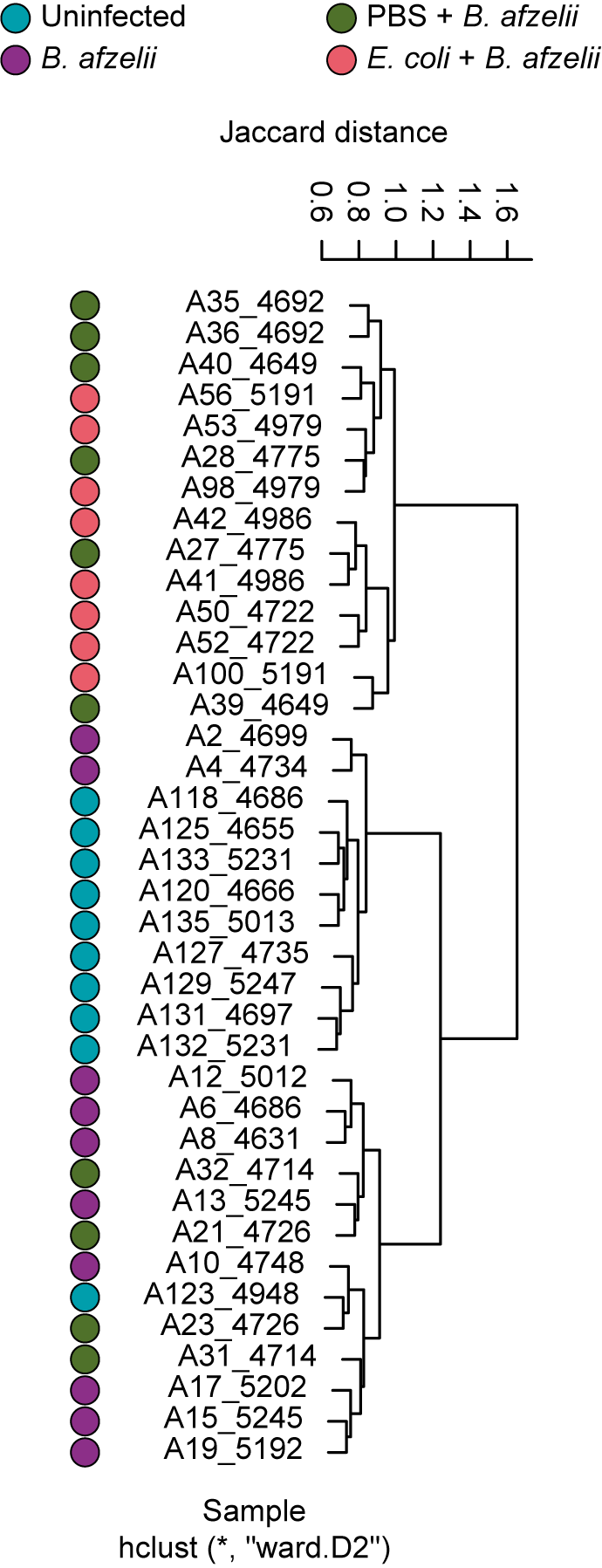

Supplement: Supplementary file 7 — Additional file 6: Supplementary Fig. S6. Cluster analysis of different samples of tick microbiota. Dendrogram based on Ward’s method of clustering for samples of tick microbiota from the uninfected, B. afzelii, PBS+B. afzelii and E. coli+B. afzelii groups. [file 40168_2023_1599_MOESM6_ESM.tif]

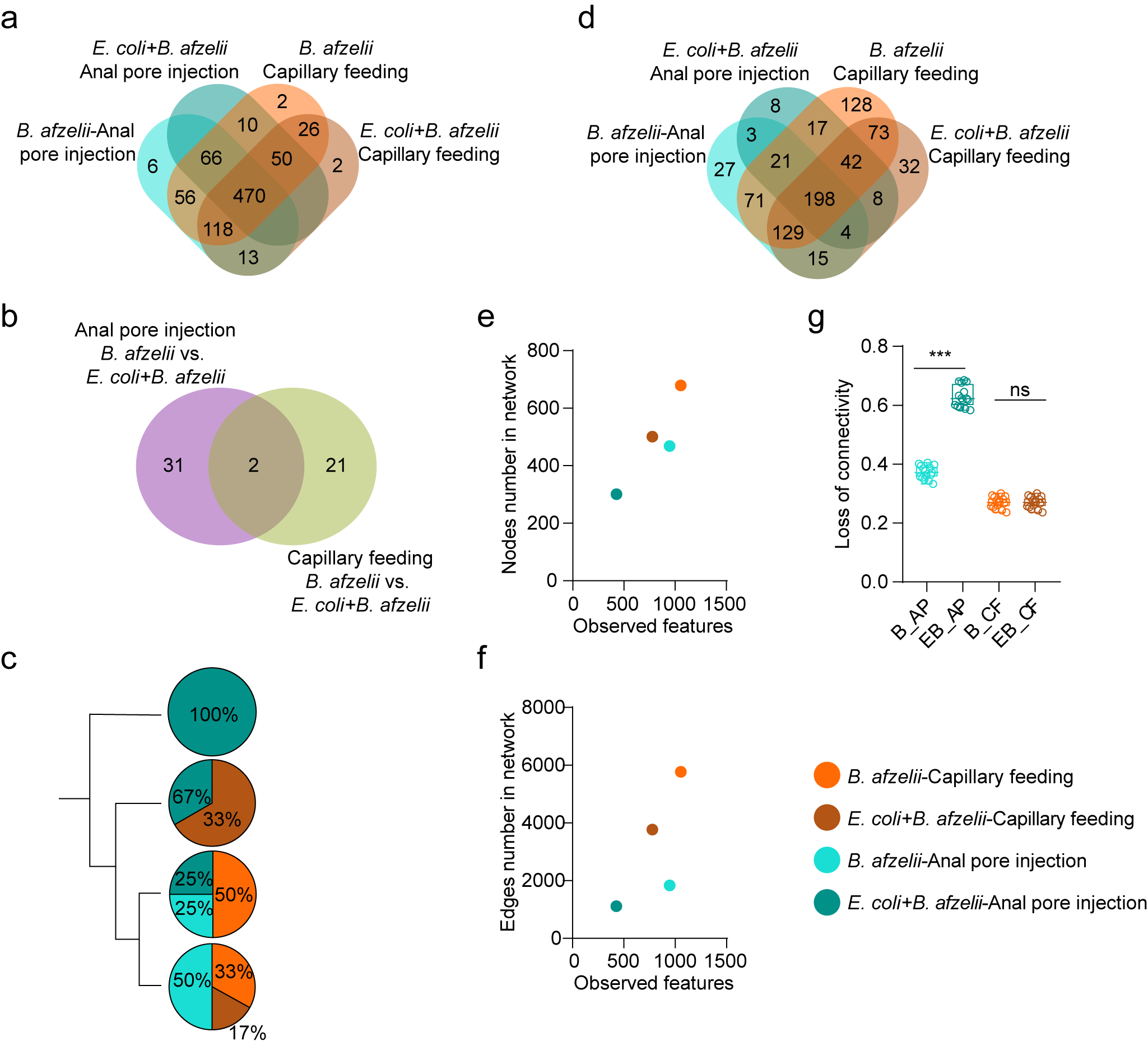

Supplement: Supplementary file 8 — Additional file 7: Supplementary Fig. S7. Impact of the addition of a commensal bacterium on the emergent properties of I. ricinus microbiota. Venn diagram showing (a) the common and unique bacterial taxa among the tick microbiota that received B. afzelii or E. coli+B. afzelii by capillary feeding and anal pore injection (b) the shared and unique taxa whose abundance changed significantly between B. afzelii vs. E. coli+B. afzelii comparisons in capillary feeding and anal pore injection groups, (c) Dendrogram of clustering for samples of tick microbiota from different experimental conditions, (d) Venn diagram showing the common and unique nodes found in microbial co-occurrence networks from all conditions. Scatter plot showing the mean of observed features versus number of (e) nodes and (f) edges found in the microbial co-occurrence networks and (g) Scatter plot showing the loss of connectivity when 5 to 7% of nodes are removed from the microbial co-occurrence network. [file 40168_2023_1599_MOESM7_ESM.tif]
